# An overview of key potential therapeutic strategies for combat in the COVID-19 battle

**DOI:** 10.1039/d0ra05434h

**Published:** 2020-07-28

**Authors:** Gaurav Das, Surojit Ghosh, Shubham Garg, Satyajit Ghosh, Aniket Jana, Ramkamal Samat, Nabanita Mukherjee, Rajsekhar Roy, Surajit Ghosh

**Affiliations:** Department of Bioscience & Bioengineering, Indian Institute of Technology Jodhpur NH 65, Surpura Bypass Road Karwar Rajasthan 342037 India sghosh@iitj.ac.in +91-291-280-1212; Organic and Medicinal Chemistry and Structural Biology and Bioinformatics Division, CSIR-Indian Institute of Chemical Biology 4, Raja S. C. Mullick Road, Jadavpur Kolkata 700 032 WB India +91-33-2473-5197 ext. 0284 +91-33-2499-5872; Academy of Scientific and Innovative Research (AcSIR) Ghaziabad 201002 India

## Abstract

The sudden ravaging outbreak of a novel coronavirus, or SARS-CoV-2, in terms of virulence, severity, and casualties has already overtaken previous versions of coronaviruses, like SARS CoV and MERS CoV. Originating from its epicenter in Wuhan, China, this mutated version of the influenza virus with its associated pandemic effects has engulfed the whole world with awful speed. In the midst of this bewildering situation, medical and scientific communities are on their toes to produce the potential vaccine-mediated eradication of this virus. Though the chances are really high, to date no such panacea has been reported. The time requirements for the onerous procedures of human trials for the successful clinical translation of any vaccine or potential therapeutics are also a major concern. In order to build some resistance against this massive pandemic, the repurposing of some earlier antiviral drugs has been done, along with the refurbishment of some immune-responsive alternative avenues, like monoclonal antibody mediated neutralization, interferon treatment, and plasma therapy. New drugs developed from the RBD domain of the virus spike protein and drugs targeting viral proteases are also undergoing further research and have shown potential from preliminary results. The sole purpose of this review article is to provide a brief collective overview of the recent status of therapeutics advances and approaches, and their current state of implementation for the management of COVID-19.

## Abstract 2

A schematic representation of different methods to inhibit SARS-CoV-2 infection.

## Graphical abstract

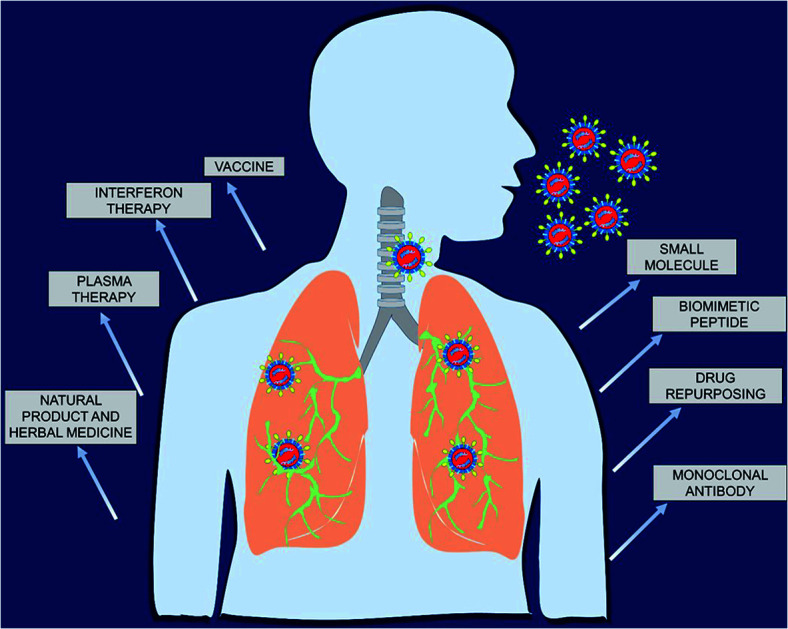

## Introduction

As COVID-19 continues to spread around the globe, researchers and drug manufacturers are moving towards the development of potential therapeutics into clinical trials at a dizzying pace. The situation has been declared an emergency by the World Health Organization (WHO) and governments across the world have given urgent consideration to controlling the transmission of this disease. Worldwide the generally accepted plan to combat this pandemic has already been taken by imposing a “lockdown” aiming to prevent the spread of virus, as SARS-CoV-2 is hugely a human-to-human contagious disease, which has also been catastrophic to the medical infrastructure of all countries. Along with governments, the people who are working as the frontline warriors in the campaign against the novel coronavirus are scientists and doctors. Researchers and medical experts are working very hard to find a promising outcome to treat this deadly disease. As we all know, viruses are non-living infectious species, which require a host cell to divide and sustain themselves. Here SARS-CoV-2, the virus causing COVID-19, is a single-stranded RNA virus, which utilizes its spike-like receptor binding domain to interact with the host cell.^1^ In the lungs, the virus targets cells expressing angiotensin-converting enzyme 2 (ACE2), which are situated in the lining of the lungs (called pneumocytes), and cause respiratory disorder.^2^ This results in a reduction in oxygen levels in the blood, which can finally lead to a fatal condition. A recent report reveals that the virus can interfere with the iron-containing compound of blood.^3^ Another study also proposes that patients with blood group A are more susceptible to SARS-CoV-2 in comparison to others.^4^ In addition, there is prevailing evidence that patients living with heart disease and diabetes are more vulnerable to this disease due to over-expression of a protein called ACE2, which SARS-CoV-2 can bind to and later use the host cell machinery for producing its duplicate copies. Hence, this further increases the rate of infection for these patients.^5^ Currently, the most generally adopted approach to treat COVID-19 infected people is to ease the patient's symptoms (which feature pneumonia), while the campaign to develop a complete cure for the disease is still a major challenge. SARS-CoV-2, being an RNA virus, can be inhibited by therapeutics used previously for curing other RNA viruses, like the Human Immunodeficiency Virus (HIV) or Ebola virus. Clinical trials are presently ongoing with a combination of two anti-HIV drugs—lopinavir and ritonavir—and also with other antiviral drugs like remdesivir.^6,7^ A concoction of several drugs, including chloroquine,^8^ a potential drug used to cure malaria, has also been repurposed for treating COVID-19 in clinical trials. Generally, a drug takes almost a decade to come to the market by succeeding in all three phases of clinical trials. Nonetheless, coordinated efforts from the governments of different countries and researchers and the availability of sufficient funds from several agencies may bring drugs against COVID-19 to the market within a restricted timeframe. In the search for therapies to treat COVID-19 at the earliest, alternatives such as monoclonal antibodies and drug repurposing are possible promising pathways, which might need less time to become available to health professionals due to their high specificity and fast clinical trials.^9^ The potency of some herbal medicines and their role in combating COVID-19 are also being studied, which function by targeting different interactions, viral enzymes, and increasing the body's immunity overall. Though no specific drugs or vaccine-mediated intervention against this deadly pandemic have yet been discovered, in this review article we are trying to recapitulate all the probable therapeutic strategies, which continue to build up some resistance and help the whole medical fraternity to fight against this major pandemic to some extent.

### Virology of SARS-CoV-2

**Fig. 1:**
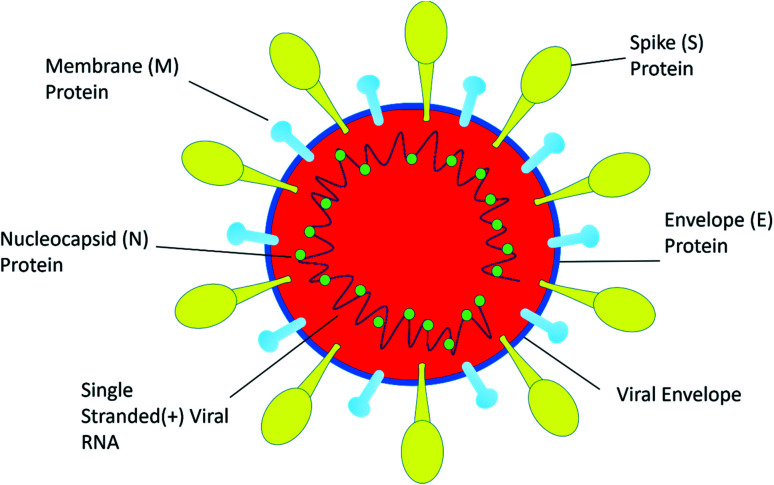
The structure of SARS-CoV-2.

SARS-CoV-2 is a single positive-stranded RNA virus of 28 to 30 kb size. SARS-CoV-2 is a mutated strain of SARS CoV, which also originated in the Guangdong province of China in 2002.^10^ SARS coronavirus contains a genomic RNA which encodes a non-structural replicate polyprotein and structural proteins, including spike (S), envelope (E), membrane (M), and nucleocapsid (N) proteins. Among all the structural proteins, the S protein is the most immunogenic, which provides protective immunity against virus infection.^11^ The membrane (M) protein and the envelope (E) protein are distributed alongside the S protein on the viral envelope (Fig. 1). To enter into the host cell the virus uses spike (S) protein, and this process requires the priming of S protein by the serine proteases of host cells, which cleaves the S protein at S1/S2 (a furin cleavage site) sites. Initially, the S1 subunit with a receptor-binding domain (RBD) helps the virus to attach to the host cell; subsequently, the S2 subunit enhances the fusion of the virus and the host cell membranes to facilitate entry.^12^ SARS-CoV-2 recognizes human ACE 2 more efficiently than SARS-CoV, thereby increasing its transmission capability from person to person. ACE 2 is a type I membrane receptor protein expressed mostly in adipose tissue, kidney, heart and small intestine.^13^ The binding affinity of RBD along with the S protein is being fully studied for vaccine development and therapeutic interventions. However, very little is known about its enteric^14^ and neurological^15^ casualties, and more research on SARS-CoV-2 would help to establish a greater accuracy in this matter.

### Therapeutic aspects of confronting COVID-19

Several options can be anticipated to control or prevent the emerging threat from SARS-CoV-2, including vaccines, peptides, small molecule drugs, monoclonal antibodies, and interferon therapies. Herein, in this section, we highlight different targets for interruption of the coronavirus life cycle both at pre-entry and post-entry stages of viral infection. These targets of the viral life cycle are mainly chosen to develop potential therapeutics to inhibit the viral pathogenesis of SARS-CoV-2.

## Small molecules as emerging potential therapeutics for inhibiting the early entry of COVID-19 by interrupting ACE2–RBD protein–protein interaction (PPI)

Based on structural and biochemical studies, it has been suggested that SARS-CoV-2 binds with human ACE2 with higher affinity than earlier SARS-CoV. As binding of virus spike protein with ACE2 is the crucial step for viral entry into the host cell, this interaction can be targeted to develop potent therapeutics against COVID-19.^16–18^ Thus, designing potential therapeutics to inhibit SARS-CoV-2 RBD/ACE2 protein–protein interaction (PPI) has been the point of interest for many researchers, as it is the first line of approach to inhibit viral entry. There needs to be a focus on small molecules with the potential to inhibit SARS-CoV-2-RBD/ACE2 complex formation without interfering with the surface expression of ACE2. The structural region in the spike glycoprotein RBD, which is mainly responsible for binding to the human ACE2 receptor, differs profoundly between SARS-CoV and SARS-CoV-2. In spite of this difference, the S proteins exhibit a sequence identity of around 76% between the two origins.^16,19^ As no new therapeutics and vaccines are available, we summarize previous efforts in developing therapeutics, which serve as entry inhibitors by blocking S protein-mediated viral attachment to the human ACE2 receptor in the case of SARS-CoV. These drugs show efficacy in inhibiting SARS-CoV-RBD/ACE2 interaction in *in vitro* studies.^20,21^ These studies will guide the design of small molecules with potency against SARS-CoV-2 S protein-mediated viral entry by further synthetic modifications of scaffolds, which have previously shown promising efficacy against SARS-CoV. Recently, the determination of the SARS-CoV-2-RBD/ACE2 complex using crystallography and CRYO-EM techniques has provided the basis for a molecular docking study in an attempt to design potent small molecules that block SARS-CoV-2 spike protein-mediated viral entry into the host cell by inhibiting PPI interaction.^19^

SSAA09E2 acts through a unique mechanism by blocking initial interactions of SARS-CoV RBD with human ACE2 receptors. The results show that SSAA09E2 does not inhibit surface expression of ACE2 by binding to an ACE2 protease active site. Thus, no disturbance in ACE2 enzymatic activity was observed, and it directly interrupts the RBD–ACE2 complex interactions.^20^ Another group of researchers has designed VE607, which also has the potential to obstruct the initial entry of the SARS CoV virus into the host cell.^21^

### Small molecules inhibiting host proteases utilized by CoVs for viral entry

The spike (S) glycoprotein of coronaviruses promotes viral entry in the host cells in two steps. Initially, the S1 subunit binds to the ACE2 receptor, which helps viral attachment to the host cell.^22^ In the next step, cellular entry requires S protein priming by the host cell proteases. This necessitates S protein cleavage at the S1/S2 fusion site, and now the S2 subunit is active for fusion with the host cell; this fusion mechanism is directed by the S2 subunit of the S protein.^23^ The presence of several arginine residues in the S1/S2 cleavage site in SARS-CoV-2, unlike that of SARS-CoV, makes the S1/S2 site more vulnerable for proteolytic cleavage and thus makes the process of viral entry more favorable by exposing the S2 subunit of the S glycoprotein. Notably, SARS-CoV-2 is thought to enter host cells *via* two distinct pathways: one moderated by serine protease TMPRSS2 at the cell surface and the other moderated by cysteine protease cathepsin L in the endosome.^24^ Accordingly, the development of protease inhibitors can be a potential target for treating COVID-19. In this regard, TMPRSS2 serine protease inhibitor camostat mesylate has already been approved as a potential therapeutic in Japan for treating chronic pancreatitis and also has antiviral properties.^25^ Markus, H. *et al.* had performed intensive *in vitro* analysis, and the results showed that clinically proven camostat mesylate could hamper the viral entry of SARS-CoV-2 similarly to that of SARS COV by inhibiting the host cell serine protease TMPRSS2. This result could be influential in the performance of further *in vivo* studies in an animal model and in humans and in this time of a global health emergency where there is urgent requirement for a drug, camostat mesylate might be a potential drug molecule clinically approved to treat COVID-19.^24^

The cellular entry of SARS-CoV-2 also shows a dependence on cathepsin L enzymatic activity *via* the endosomal pathway, so cathepsin L inhibitors were also identified as a novel target to inhibit COVID-19 entry into the host cell.^24^ Herein, we highlight some of the potent cathepsin L inhibitors which show least interference with the cleavage of a host protein-derived peptide (pro-neuropeptide Y) such as SSAA09E1. Several thiourea derivatives such as SSAA09E1 have formerly been shown to have potent antiviral properties against HIV and hepatitis C virus. SSAA09E1 is a non-peptidomimetic small molecule that inhibits cathepsin L activity.^20^ Yanchen, Z. *et al.* reported the development of K11777, which had antiviral activity by targeting cathepsin L mediated cell entry. K11777 is already in the forward stages of development for treating several parasitic diseases and has proven to be effective and nontoxic in a wide range of animal models. K11777 has proven efficacy for inhibiting SARS CoV and Ebola with an IC_50_ value in the sub-nanomolar range. *In vitro* studies suggest that when K11777 and serine protease inhibitor camostat mesylate are administered together, they show greater efficacy for the complete inhibition of SARS CoV entry.^26^

The potential of glycopeptide antibiotics for preventing cellular entry of the Ebola virus, SARS-CoV, and MERS CoV by inhibiting the activity of cathepsin L was previously known. The potency of these glycopeptide antibiotics is now being evaluated for treating SARS-CoV-2, and notably it has been found that teicoplanin has a potent ability to prevent SARS-CoV-2 virus entry with an IC_50_ of 1.66 μM. Although *ex vivo* and *in vivo* studies need to be done, the preliminary idea of this study suggests that the antiviral activity of teicoplanin might be applied to treat SARS-CoV-2 viral infection in the host cell.^27^

The novel approach presented in this study suggests that cathepsin L inhibitors, administered in combination with clinically approved serine protease TMPRSS2 inhibitor camostat mesylate, show significant improvement in efficacy for complete inhibition of viral entry into a host cell for all types of S glycoprotein based virus, including COVID-19. Small molecules with cathepsin L inhibiting activity can be further optimized and developed into a broad range of potent antiviral therapeutics, with high specificity in their activity and thus prevent viral entry without affecting the normal ability of proteases to process host proteins, while the clinically approved drug camostat mesylate can immediately answer current purposes in a global pandemic where the development of new drugs and their clinical approval is time-consuming.

Recently a group of researchers suggested that a unique furin-like cleavage site is present in the spike glycoprotein of SARS-CoV-2, which is missing in the other ranges of SARS-like CoVs. In their article, the group focussed on a peculiar furin-like protease recognition sequence present in close proximity to one of the maturation sites of the S protein that might have convincing functional implications for viral entry.^28^ The molecular mechanism involved in cellular entry by activation of the S protein has not yet been conclusively identified and needs further evaluation. Thus, it is likely that, apart from the other two host cell proteases (TMPRSS2 and cathepsin L) involved in priming of S glycoprotein into the S1/S2 subunit, furin might also be involved in this cleaving mechanism, thereby enhancing viral fusion with the host cell. The furin-like cleavage site might play a significant role in the viral life cycle of SARS-CoV-2. A noteworthy feature of SARS-CoV-2 is a polybasic cleavage site at the terminal of the S1 and S2 subunits of the S glycoprotein, and this further allows effective cleavage by furin and other proteases and has a significant role in viral entry into host cells.^29–31^ Thus, the drive to develop anti-COVID-19 therapeutics should also involve evaluation of furin inhibitors. The pathogenesis of some previously occurring coronaviruses has been reported to be dependent on a furin-like cleavage site in the S-protein sequence. Thus therapeutics developed for those viruses can be evaluated to test their efficacy against SARS-CoV-2. In this line of approach, a group of researchers has developed peptide-based therapeutics to irreversibly block the enzymatic activity of furin protease by the addition of a decanoyl group at the N-terminus and a chloromethyl ketone (CMK) group to the C-terminus of a polybasic cleavage motif to favor cell penetration (dec-RVKR-cmk).^32,33^ As furin-like protease is involved in multiple cellular processes, so specific inhibition is a major challenge that may result in some toxicity.^28^

As the cleavage of the S protein into its two subunits S1 and S2 is necessary for viral entry, the involvement of different host cell proteases in activating this S glycoprotein needs to be studied thoroughly. Thus, the potential for furin inhibitors to halt SARS-CoV-2 pathogenesis in *in vitro* and *in vivo* studies needs to be evaluated, so that in the campaign to develop potent therapeutics to treat COVID-19, we might consider the presence of furin inhibitors.

### Therapeutics inhibiting the viral proteases 3CLpro and PLpro

The typical coronavirus (CoV) is a single-stranded positive-sense RNA virus that possesses a large viral RNA genome. The two open reading frames 1a/b (ORF1a/1b) at the 5′ terminal enclose the 5′ two-thirds of the CoV genome and encompass the large replicase polyproteins 1a (pp1a) and pp1b. These polyproteins are chopped by papain-like cysteine protease (PLpro) and 3C-like serine protease (3CLpro) viral enzymes to deliver non-structural proteins, which include RNA-dependent RNA polymerase (RdRp) and helicase, and are engaged in the process of transcription and replication of the virus.^34^ Therefore, inhibiting 3CLpro and PLpro activity is a potential target for treating CoVs.

Sequence and structural analysis suggest that when the 3CLpro protein sequence of SARS-CoV-2 was correlated with its closest homologs, it showed a 99.02% sequence identity with bat SARS-like coronaviruses and 96.08% with SARS-CoV. Recent studies show that the genome sequence of SARS-CoV-2 is very similar to that of SARS-CoV.^35–37^ The results also proclaim that the SARS-CoV-2 and SARS-CoV 3CLpro receptor binding pocket conformations resemble each other, thus raising the possibility that drugs designed to inhibit the 3CLpro of SARS-CoV can also be utilized to inhibit the 3CLpro of SARS-CoV-2.^38^ Different classes of protease inhibitors can target SARS-CoV, and these 3CLpro inhibitors show a broad range of *in vitro* action opposing CoVs.^39^ Amid the distinct collection of 3CLpro inhibitors, lopinavir is readily available. Lopinavir, a protease inhibitor employed to cure HIV disease that has been clinically approved as a ritonavir-supported form (lopinavir–ritonavir) inhibits the HIV protease enzyme by constructing an inhibitor–enzyme complex, thereby forbidding cleavage of gag-pol polyproteins. Thus, immature, non-infectious viral particles are formed subsequently. Ritonavir increases lopinavir's pharmacodynamic and pharmacokinetic activity by slowing down the breakdown of lopinavir. This lopinavir–ritonavir combination showed *in vitro* anti-CoV activity; also *in vivo* analysis with a MERS CoV infected animal model and non-randomized clinical trials in SARS patients exhibited properties inhibiting 3CLpro protease activity. It is speculated that the lopinavir–ritonavir 3CLpro inhibiting activity contributes partly to its anti-CoV effects.^40,41^ Although the efficacy of this combination of the drug was not associated with clinical improvements when clinical trials were performed with SARS-CoV-2 infected patients.^42^ These results of a clinical trial suggest that it is necessary to discover novel compounds with inhibitory properties against SARS-CoV-2 3CLpro enzyme activity to serve as potent anti-COVID-19 drugs. Recently, drug repurposing studies by Zhijian, X. *et al.* have proposed that the clinically approved antiretroviral drug nelfinavir, used in the treatment of HIV, shows better efficacy against SARS-CoV-2 3CLpro activity and suggest that it could be used to treat COVID-19.^43^ Linlin Zhang and his co-workers in recent times have reported the X-ray structure of the unliganded SARS-CoV-2 3CLpro and its complex with an α-ketoamide inhibitor. Optimization of α-ketoamides as 3CLpro inhibitors proved crucial to blocking viral replication. They developed a potent lead compound 13b derived from a previously designed inhibitor that inhibits SARS-CoV-2 3CLpro activity with IC_50_ = 0.67 ± 0.18 μM. An EC_50_ of 4–5 μM was observed when human Calu3 cells were infected with SARS-CoV-2. After assessing the adsorption–distribution–metabolism–excretion (ADME) properties of the lead compound, the group suggested that development of the pyridone-containing α-ketoamides inhibitors might have efficacy against the 3CLpro activity of SARS-CoV-2.^48^

Although the 3CLpro conformations of SARS-CoV and SARS-CoV-2 resemble each other, they show some key differences.^38^ Blocking the activity of 3CLpro will inhibit viral replication, and also 3CLpro inhibitors are unlikely to be toxic as no human proteases have a similar cleavage specificity. Thus, there is a specific demand for a drug to inhibit 3CLpro activity of SARS-CoV-2.

Plpro protease is unique in its nature as this protease is not only capable of processing the viral polyproteins into the structural and non-structural protein components essential for viral replication, but it is also responsible for deubiquitinating host cell proteins such as interferon regulatory factor 3(IRF3), inactivation of the transcription factor NF-κB and has deISGylating activities, and thus plays an important role in suppressing the human immune system.^44,45^ Unlike 3CLpro, the PLpro of SARS-CoV and SARS-CoV-2 do not resemble each other, their two origins sharing only 83% sequence identity. However, the active sites of PLpro protease from two different origins show no variation in three secondary structures.^36,37^ Thus, it is possible that SARS-CoV PLpro inhibitor drugs might also show efficacy against SARS-CoV-2 PLpro. There has been extensive research going on into the development of efficient small molecules to inhibit PLpro activity, and these compounds display efficacy in the μM range, thus opening the window for the further development of novel small molecules to inhibit PLpro activity. Here we highlight some of the work by a group of researchers in developing potent PLpro inhibitors. Kiira, R. *et al.* developed a noncovalent class of PLpro inhibitors, and the resulting compound GRL0617 shows efficacy against SARS-CoV with an IC_50_ value of 20 μM, which was improved to the nanomolar range *via* synthetic optimization. GRL0617 shows no associated toxicity and inhibits SARS-CoV viral replication in *in vitro* studies. These findings suggest that noncovalent cysteine protease inhibitors can be developed with high specificity against the processing of viral polyproteins without inhibiting host deubiquitinating enzyme.^46^ Another group of researchers had identified a lead compound 6577871 *via* high-throughput screening of a diverse chemical library, and its further synthetic optimization and structure–activity analysis were performed to generate a library of improved inhibitors that show potent PLpro inhibition and antiviral activity against SARS-CoV infected host cells. These studies show a substantial increase in the efficacy of the small molecules with a nanomolar range. Further protein-ligand X-ray structure, molecular modeling, and biological evaluation of a series of PLpro inhibitors provide molecular insight into the ligand-binding site interactions.^47^ Apart from these, various SARS-CoV PLpro inhibitors were analyzed by Báez-Santos and co-workers, which include small molecule inhibitors, natural products, zinc ion and zinc conjugate inhibitors, thiopurine compounds and naphthalene inhibitors.^44^

**Fig. 2:**
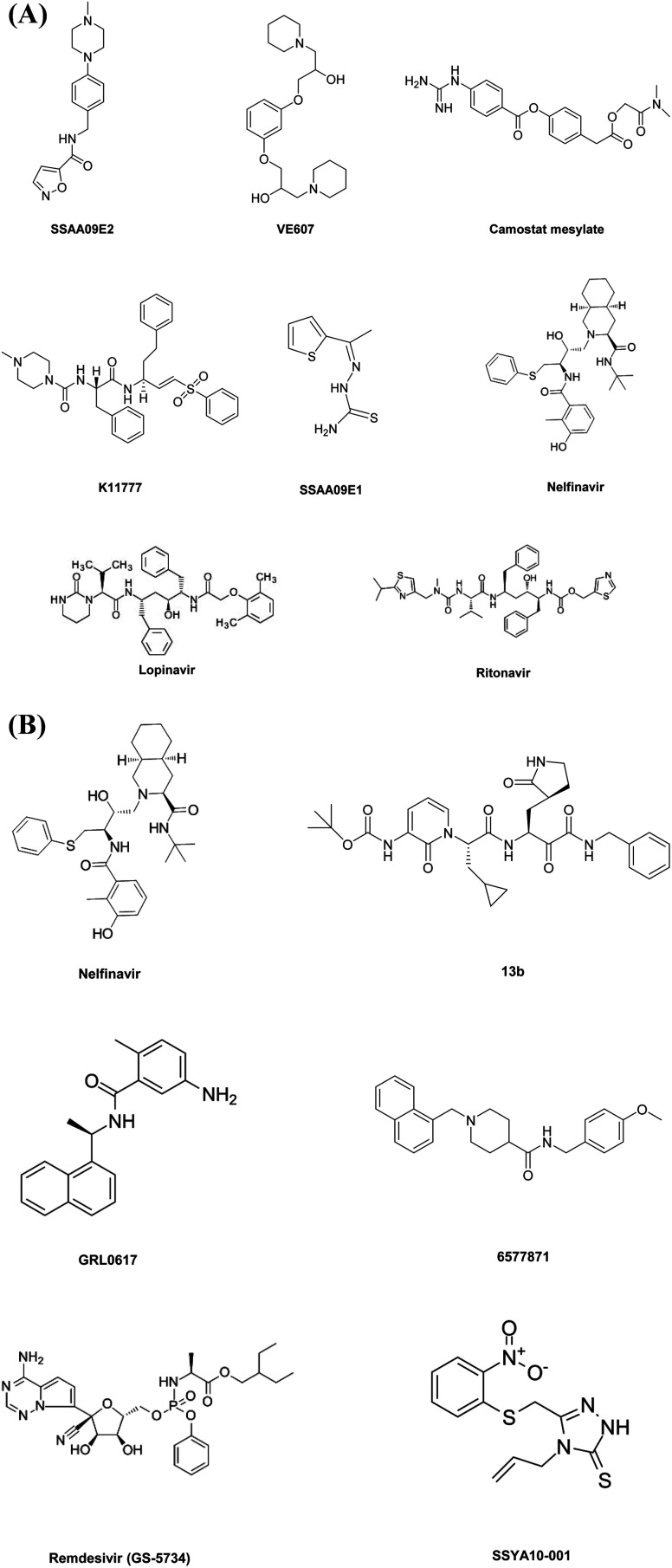
(A and B) Small-molecule-based drug structures.

This diverse chemical library of novel therapeutics with potent anti PLpro activity opens room for the further development of drugs with specific inhibitory capacity against SARS-CoV-2 PLpro activity. Fig. 2A and B include the structures of the small molecules discussed in the section on small molecule based therapeutics to combat COVID-19.

## The inhibition of viral replication enzymes can also be a potential target to treat COVID-19

Polyproteins from coronavirus genomes after being processed by viral enzymes 3CLpro and PLpro form non-structural proteins, which include RNA-dependent RNA polymerase (RdRp) and helicase (Hel), and take part in the process of transcription and replication of coronavirus by using the host cell's machinery. Thus, inhibition of this viral replication process could be a potential target for the control of viral infection. RdRp proteins share 96% sequence identity between SARS CoV and SARS-CoV-2, although their genomic RNA shows an 82% sequence identity. Residues that show variations in RdRp proteins between two coronaviruses are mostly far away from the active site.^49^ This high sequence identity at the active site of the RdRp protein between two coronaviruses suggests that any potent therapeutics developed for SARS-CoV RdRp might show equal potency against SARS-CoV-2 viral replication. RdRp inhibitors are mainly nucleoside or nucleotide analogs, and they provide the most assured pathway towards inhibiting viral RNA replication. The nucleoside analog ribavirin with broad-spectrum antiviral properties has been previously used to treat a viral infection, but its efficacy against RNA viruses, including coronaviruses was unclear.^50,51^ The efficacy of these nucleoside analog drugs can be increased by inhibiting exonuclease activity by the enzyme non-structural protein (nsp14). This nucleoside analog drug functions by insertion into the viral RNA chains causing their premature termination, whereas, in contrast, nsp 14 enzymatic activity has a proofreading ability and thus complicates the antiviral nucleoside drug objective. The nsp14 has the ability to remove mismatches as well as to abolish nucleoside analogs which were incorporated into the viral RNA chain. Thus, in order to develop a potent nucleoside analog drug, it must either avoid detection by exonuclease or must inhibit exonuclease activity.^52,53^

Remdesivir (GS-5734) is a magnificent illustration of this approach, as this drug show a promising outcome against the RdRp activity of viral RNA replication by outcompeting exonuclease activity. It is an adenosine analog with a 1′-nitrile substituent which exhibits potent efficacy against SARS, MERS, and BAT coronavirus in *in vivo* and *in vitro* studies.^54,55^ Remdesivir shows potential against a broad spectrum of coronaviruses, and thus it is being studied for post-infection treatment for COVID-19. Wang *et al.* displayed data showing that remdesivir is active against SARS-CoV-2 in Vero E6 cells with an EC_50_ of 0.77 μM, suggesting its ability to be a potential drug candidate to fight COVID-19.^56^ Furthermore, *in vivo* studies in an animal model infected with SARS-CoV-2 was found to prevent disease progression with remdesivir.^57^ Clinical trials in the USA and China show an improvement in the patient's condition when treated with remdesivir, but no broad conclusion can be made based on a few clinical trials. Thus, further research is needed before a conclusion can be drawn.^56,58^

Apart from remdesivir, many other nucleoside analogs, including DNA synthesis inhibitors such as tenofovir, disoproxil, lamivudine, and similar other antiviral medications, have the potential to target the SARS-CoV-2 viral RNA replication mechanism, when evaluated through molecular docking studies and testing in cells infected with the virus.^59^ Thus, drug repurposing studies on existing nucleoside analogs can help to combat the global health emergency of COVID-19 and also open the door for further synthetic modifications of these drugs, which could help to generate a broad spectrum of anti-viral therapeutics useful for future coronavirus-related outbreaks. During the coronavirus replication cycle, helicase catalyzes the unwinding of double-stranded oligonucleotides into single strands in an ATP-dependent reaction. Helicases of different coronaviruses are highly homologous in their sequence identity. Thus, the development of potent therapeutics to inhibit helicase activity are also an attractive option to treat coronavirus pathogenesis.^60^ Coronavirus helicase inhibitors can be widely classified into two groups based on their mechanism of action. The first group includes bananins and 5-hydroxychromone derivatives, which show efficacy in hindering the unwinding process and ATPase function of SARS-CoV helicase in *in vitro* studies, which results in the inhibition of viral replication by using the host cell's machinery.^61,62^ However, this group of compounds exhibits toxicity resulting from the blockage of cellular kinases or ATPase, restricting their use for humans. The other class of coronavirus helicase inhibitors includes a compound that specifically hinders the unwinding activity without hampering the ATPase function of CoV helicase. In this line of approach, Adedeji, A. O. *et al.* have designed a triazole SSYA10-001, which was specific in its activity to inhibit a broad spectrum of coronaviruses, including SARS-CoV and MERS-CoV.^63,64^

**Fig. 3:**
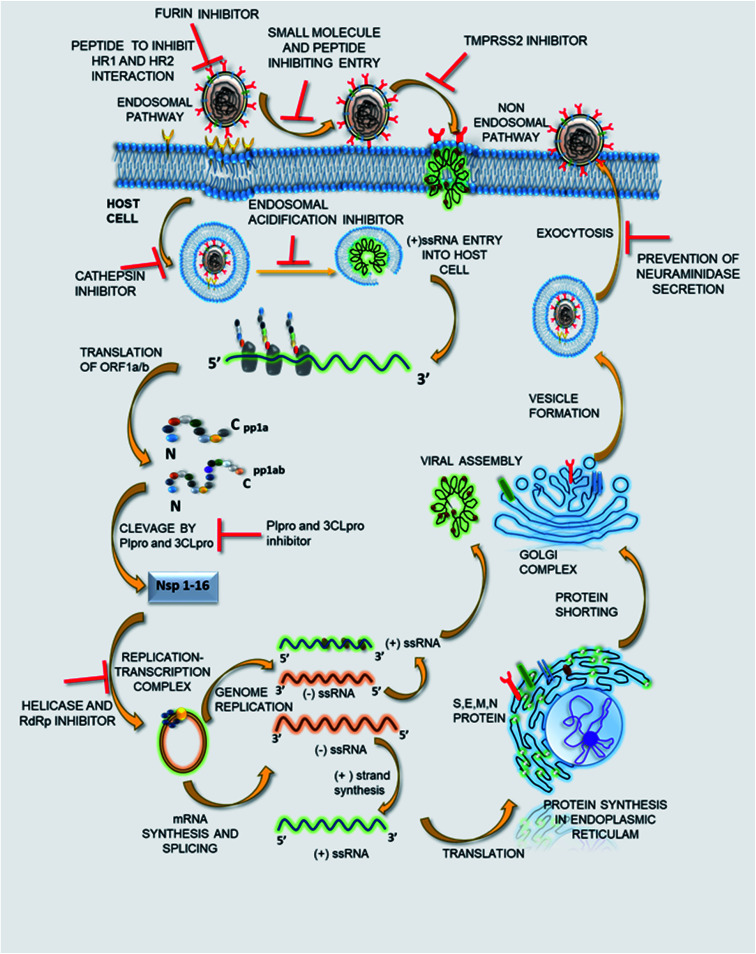
The viral life cycle and potential therapeutic targets.

The potential of SSYA10-001 and its further synthetic modifications to develop a small molecule library inhibiting SARS-CoV-2 viral replication in *in vivo* and *in vitro* studies needs to be evaluated. Thus, it might be that potent therapeutics with a broad spectrum of antiviral properties could be used in combating COVID-19 and such coronavirus-related outbreaks in the near future. Fig. 3 represents the viral life cycles and potential therapeutic targets that can be used to treat COVID-19 infection. The list of drugs to deal with the same is given in Table 1.

**Table 1:** Target-based therapeutics of SARS-COV-2

Targeted host factors/viral component for interrupting the virus life cycle	Examples	Mechanism of action	Comments	References
(1) Viral spike protein with human ACE2 interaction	(A) Small molecule		Small molecule and peptides are capable of preventing viral entry either by breaking the interaction or by inhibiting viral cell fusion with the host cell	20 and 21
	1. SSAA09E1	1. Effective if applied 1h post infection by breaking the interaction		
	2. VE607	2. Blocks S1 protein RDB–ACE2 mediated cellular entry		
	(B) Peptide based			70 and 73
	SBP1, inhibitors (1–4)	Have identical sequences of amino acids to ACE2 and binds with the S1 portion of the viral spike protein		
(2) Host proteases utilised by SARS-CoV-2 for viral entry			Observed good results when a cathepsin L inhibitor is administered with a serine protease inhibitor; a significant increase in efficacy is observed in preventing viral cell entry	
(A) Cysteine protease cathepsin L	(A) SSAA09E1, K1777, glycopeptide *e.g.* teicoplanin	(A) Inhibits S1/S2 cleavage at the tested concentration		20, 26 and 27
(B) Serine protease TMPRSS2	(B) Camostat mesylate	(B) Inhibits host cell serine protease and prevents the cleavage of S1/S2		24 and 25
(C) Other host proteases (furine)	(C) dec-RVKR-cmk	(C) A furin inhibitor that blocks the furin mediated cleavage of the S protein		33 and 34
(3) HR1 and HR2 interactions at the S2 subunit of the spike glycoprotein	Peptide EK1C4	The peptide derived from HR2 binds with HR1, and a native complex cannot form	Further modification of biomimetic peptides and *in vivo* efficacy in animal models can support the development of peptide based therapeutics	80
(4) Viral enzymes				
(A) PLpro	(A) GRL0617, 6577871	(A) Inhibits PLpro activity	(A) Narrow spectrum	46 and 47
(B) 3CLpro	(B) Lopinavir–ritonavir, nelfinavir, 13b	(B) Inhibits 3CLpro activity	(B) Ritonavir increases lopinavir pharmacodynamic and pharmacokinetic activities when used together	40, 41, 43 and 48
(C) RNA dependent RNA-polymerase (RdRp)	(C) Remdesivir (GS-5734), ribavirin	(C) Nucleoside analog drugs function *via* inserting into viral RNA chains, causing their premature termination, and they are also used for post-infection treatment	(C) Active against SARS and MERS coronaviruses in *in vitro* studies at high doses	50, 51, 54–57 and 59
(D) Helicase	(D) SSYA10-001	(D) Inhibits helicase unwinding and ATPase activities	(D) Inhibits the activity of a broad range of corona viruses	61–64

## Biomimetic peptide based therapeutics to inhibit SARS-CoV-2 viral attachment to the ACE2 receptor

**Fig. 4:**
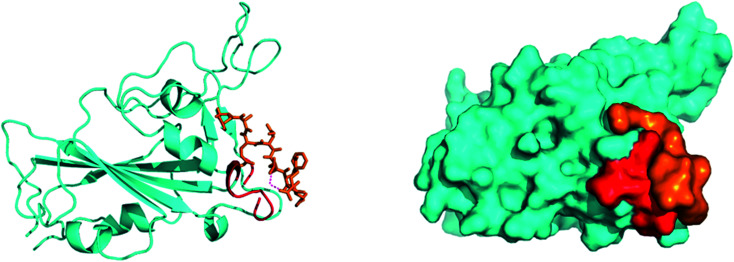
Preliminary blind docking data revealed the interacting stretch of amino acids of the ACE2 receptor (red) with the viral spike RBD (cyan): the ‘Hotspot’ zone.

SARS-CoV-2, similar to the earlier SARS-CoV, initiates entry into the human cell by binding to angiotensin-converting enzyme (ACE2). The interaction of the SARS-CoV-2 spike glycoprotein receptor-binding domain (RBD) with the ACE2 receptor is the crucial step in viral attachment to the host cell.^65^ This is followed by S protein priming by the host cell proteases that are required for viral entry into the host cell and subsequent viral replication *via* viral proteases by using host cell machinery.^66^ Based on recent studies, it was suggested that SARS-CoV-2 binds with the ACE2 receptor with higher affinity than the earlier SARS-CoV.^65^ Thus, disrupting the SARS-CoV-2 RBD/ACE2 complex is the most promising target to block the viral entry in the very first stage of its attachment to the human receptor ACE2 at the surface of the host cell^67^ (Fig. 4).

As SARS-CoV-2-RBD/ACE2 protein–protein interactions (PPI) cover large protein binding interfaces, small molecule based inhibitors lacking distinct binding pockets are often less useful in preventing this interaction.^67^ Thus, an approach to designing peptide-based antagonists of the human ACE2 receptor is an exciting solution to hinder RBD–ACE2 interaction by appropriately covering the extended protein contact interface.^68,69^ In this line of approach, by employing molecular dynamics simulations on the basis of the recently examined co-crystal structure of ACE2 and the SARS-CoV-2-RBD complex, researchers have identified a stretch of crucial helical amino acid sequence (α1 helix) at the ACE2 peptidase domain, which is important for binding SARS-CoV-2-RBD. Towards this aim, the group has designed a 23-mer peptide fragment (SBP1) of the ACE2 PD α1 helix, a sequence of which was derived from the ACE2 α1 helix. This peptide binder binds specifically with low nanomolar affinity with the SARS-CoV-2 RBD. Thus, this peptide binder opens up new ventures for SARS-CoV-2 treatment by blocking the spike glycoprotein interaction with ACE2. The interaction between SPB1 and the RBD of the SARS-CoV-2 spike protein was further validated by bio-layer interferometry, which suggests that an SPB1 peptide derived from the ACE2-PD α1 helix may efficiently bind the SARS-CoV-2 spike protein and outcompete the binding for ACE2.^70^

Another group of researchers have recently designed a group of peptide-based inhibitors based on the latest knowledge of the protease domain (PD) of ACE2 that involves mainly the α1 helix with minor involvement from the α2 helix and the linker of the β3 and β4 sheets, which binds effectively with the RBD of the SARS-CoV-2 spike protein.^67,71^ In the previous work on a SARS-CoV blockade, small peptide inhibitors were proposed, which were too short to maintain the secondary structure to block the whole SARS-CoV binding surface.^72^ By analyzing the crystal structure of ACE2 and the RBD of SARS-CoV-2, interacting amino acids were identified at the ACE2 and RBD interface. Therefore, the group has designed four inhibitors (inhibitors 1–4) for four different critical binding hotspots, mainly inhibitor 1 for the vital α1 helix. The report further suggests that the single α1 helix used in inhibitor 1 is less balanced, while the α1,2 helices used in inhibitors 2–4 backed each other and preserved their bent shape, which contributes to the conformational identity of the RBD of SARS-CoV-2, and a full envelope of the RBD surface.^73^

Thus, the designed peptides derived from the human ACE2 hotspots which bind to the RBD of the SARS-CoV-2 spike protein provide room for the further development of peptide-based therapeutics for treating COVID-19.^70,73^ During a pandemic such as COVID-19, therapeutic intervention is needed urgently, and in this regard, peptide-based therapeutics are promising alternatives because of their high specificity, low interference with biological processes, and faster FDA approval times.^74^ Further optimization of these peptide-based therapies is in progress to significantly increase their PPI inhibitory activity. Challenges associated with peptide-based drugs, such as rapid renal elimination and proteolytic degradation, need to be figured out in the near future to develop potent peptide-based therapeutics to inhibit the viral entry of SARS-CoV-2 and such a broad spectrum of coronaviruses.

## The inhibition of SARS CoV-2 by developing potent peptide based inhibitors targeting HR1–HR2 interaction at the S2 protein of the coronavirus

**Fig. 5:**
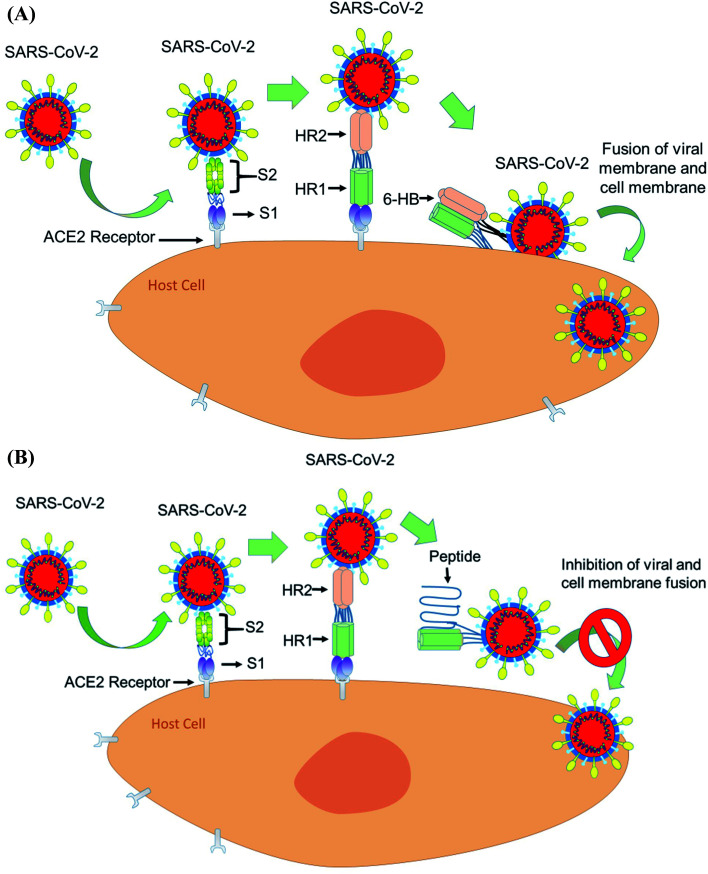
(A) The mechanism of interaction between ACE2 and the S subunits of SARS-CoV-2 to facilitate the entry of the virus into the cells. (B) The inhibition of SARS CoV-2 by developing potent peptide-based inhibitors targeting the HR1–HR2 interaction at the S2 protein of the coronavirus.

The spike glycoprotein of coronavirus has two subunits S1 and S2. The S1 subunit binds the cellular receptor with its receptor binding domain (RBD), whereas the S2 subunit has a significant role in membrane fusion and viral entry to the host cell. When S1 binds to human ACE2, followed by a conformational change in the S2 protein, the heptad repeat 1 (HR1) comes into close contact with the heptad repeat 2 (HR2) within S2. Now, the HR1 and HR2 domains of the S2 protein bind to each other to form a six-helical bundle (6-HB) core structure and this mechanism allows the viral and cellular membrane to come into close proximity for fusion^75^ (Fig. 5A).

From the sequence alignment studies, the SARS-CoV and SARS-COV-2, S2 subunits are highly preserved. The HR1 and HR2 domains have 92.6% and 100% identities, respectively.^76^ As the HR2 domain sequence is identical for both SARS CoV and SARS-CoV-2, the recently emerged coronavirus SARS-CoV-2 can be inhibited by a similar approach to that used for SARS CoV fusion inhibition by designing HR1- and HR2-derived peptides. Shuai, X. *et al.* have concluded that the SARS-CoV-2 HR1 and HR2 domains are also able to interact similarly to that of SARS-CoV to form the 6-HB core and suggest that a biomimetic peptide based on HR1 and HR2 peptide domains can inhibit COVID-19 fusion entry into the human host cell^77^ (Fig. 5B).

As the RBD of the S1 protein which interacts with human ACE2 receptors under the evolution process becomes highly mutated in different coronaviruses, this cannot be the ideal target to treat a wide variety of viruses of this family. Whereas, in contrast to the mutated S1 protein, the HR domain of the S2 protein is preserved in various human CoV viruses and plays a key role in human CoV infections by forming a 6-HB core that mediates viral entry into the human host cells.^78^ Based on experimental findings, it was found that HR1 is the target site and the HR2-derived peptide (HR2P) inhibits the 6-HB core formation in a similar way to that in the case of SARS CoV. Interestingly pan-coronavirus fusion inhibitor EK1, designed previously to inhibit the viral entry of SARS CoV by targeting the HR1 domain, also shows significant inhibitory activity with an IC_50_ of 0.19 μM by binding to the HR1 domain of the S2 protein for SARS-CoV-2, though the HR1 domains of SARS-CoV and SARS-CoV-2 show significant sequence variation.^79^

Recent studies have suggested that the mutated HR1 domain of the S2 protein in the case of SARS-CoV-2 leads to more efficient interaction with the HR2 domain to form the 6-HB core. So modified EK1 pan coronavirus inhibitors were developed, such as EK1C4 which has more potent inhibitory activity against COVID-19 than the original EK1 peptide. This EK1C4 was designed based on numerous reports on lipidation strategy that can significantly improve the antiviral property of fusion inhibitory peptides. This lipopeptide hybrid molecule EK1C4 has potent inhibitory activity against viral entry with an IC50 of 1.3 nM, suggesting that EK1C4 has potent COVID-19 inhibition properties with no toxic effects in *in vitro* studies. EK1C4 also displays a broad spectrum of inhibitory effects against other human coronaviruses. Therefore, EK1C4 when administered intranasally is expected to have potent therapeutic activity against SARS-CoV-2 infection.^80^

Thus further modification of this biomimetic peptide and its *in vivo* efficacy in the animal models could open up a window for the development of potential peptide-based therapeutics against COVID-19 and other such future emerging coronavirus-related epidemics.

## Potential small molecule based drugs repurposed to treat COVID-19

With new interventions, it may take a long time for researchers to develop and commercialize these therapeutics to treat COVID-19. Thus, to combat this pandemic at the earliest, drug repurposing studies with available drugs have the advantages of easy availability from the pharmaceutical supply chain and also being clinically approved.

(1) Chloroquine and hydroxychloroquine (HCQ) are clinically approved antimalarial and autoimmune disease drugs that can be utilized as broad-spectrum antiviral drugs.^82^ Both drugs are known to block viral pathogenesis in two stages. At the entry point, they block the viral infection by increasing endosomal pH, and another antiviral mechanism is related to glycosylation inhibition of newly synthesized proteins in the post-entry stages of viral pathogenesis. Apart from their antiviral activity, chloroquine and HCQ also have an immune-modulating role, which enhances the antiviral efficacy of the drugs *in vivo*.^83^ HCQ is already being used in clinical trials to treat AIDS (NCT01067417). Both chloroquine and HCQ can improve the clinical results of patients infected with SARS-CoV-2. HCQ appears to be safer and more potent in inhibiting the SARS-CoV-2 virus *in vitro*. HCQ also has comparatively fewer side effects than chloroquine.^84,85^ A recent clinical trial with patients infected with SARS-CoV-2 virus suggested that when HCQ is administered in combination with macrolide antibiotic azithromycin it showed a better clinical outcome in patients compared to those treated with HSQ alone.^86^ The shortage of any conclusive statement and growing controversy regarding the application of HCQ for improving the condition of SARS-CoV-2 infected patients have driven the scientific community to undertake more randomized clinical trials. Thus, its further validation is needed, which can help to provide clinical guidance on the use, dosage of the drug, and its potential in treating COVID-19.

(2) Oseltamivir is a clinically approved drug for treating influenza viruses. It belongs to the class of viral neuraminidase inhibitors. This drug has proved its potency in preventing the spread of the influenza virus by inhibiting the activity of viral neuraminidase enzyme.^87^ Viral neuraminidase is a type of neuraminidase found on the surface of the influenza virus that cleaves the sialic acid from host cell glycoproteins and enables the reproduced virus to be released from the host cell. In the influenza virus, neuraminidase facilitates the mobility of the virus to and from the site of infection.^84,88^ Thus, viral neuraminidase inhibitor drugs display potency against a broad range of influenza viruses. The use of oseltamivir was reported in a clinical trial to treat COVID-19 either with or without antibiotics and corticosteroids.^89^ Adaption of oseltamivir with combinations of other drugs like chloroquine and favipiravir was also reported in a clinical trial. Such a combination of drugs has exhibited potency against SARS-CoV-2 with an EC_50_ value of 61.88 μM and low toxicity (NCT04303299).

(3) Arbidol, also familiarly known as umifenovir, is already clinically approved in China and Russia for the cure of influenza viral infections. The arbidol antiviral mechanism against the influenza virus involves inhibition of viral fusion with the cellular membrane, which restricts viral entry into the host cell.^90^ Some reports suggest that the drug is more active against RNA viruses than against DNA viruses.^91^ Apart from its antiviral properties, arbidol also exhibits immune-modulating activity. Notably, arbidol has no detrimental effects and has been utilized for SARS treatment.^89^ Clinical trials are being conducted with arbidol for COVID-19 treatment, in comparison with the basic treatment (NCT04260594). This drug is also administered in combination with other well-known antiviral drugs, which inhibit viral pathogenesis by a different mode of action to treat patients infected with SARS-CoV-2 (NCT04255017 and NCT04252885). The clinical outcome shows an overall virus-negative conversion rate (NCT04260594).

(4) Nucleoside analogs are inhibitors of viral enzymes RdRp. These drugs show a broad spectrum of antiviral activity against RNA viruses. Clinically approved nucleoside analogs (favipiravir and ribavirin) and experimental nucleoside analogs (remdesivir and galidesivir) might have potential for treating COVID-19.^81^ The mechanism of action by nucleoside analog drugs to inhibit viral replication is briefly described in this review under the section on RdRp inhibitors. A clinical trial of remdesivir has already started to treat patients infected with COVID-19. Similarly, for favipiravir, a pyrazine carboxamide derivative already in use in Japan to treat the influenza virus is being tested for evaluating its clinical efficacy against COVID-19 (NCT04319900).

(5) Remdesivir, which was an earlier reported drug for treating Ebola also shows promising efficacy in initial clinical trials to treat COVID-19. Containing mono phosphoramidate functionality, remdesivir, which is also known as GS-5734, is a prodrug of an adenosine analog and possesses significant effectivity against a diverse spectrum of viruses, including pneumoviruses and coronaviruses. Its major metabolic byproducts interfere with the viral RNA polymerase activity, thereby lowering its replication in the host cell. But further studies, with a larger number of patients, are needed to validate these results^85^ (NCT04257656).

(6) Famotidine is an antacid. Reports from China and molecular modeling results suggest that famotidine could make a difference in confronting COVID-19; the drug seems to bind a key enzyme PLpro in SARS-CoV-2. Delivery of this drug for COVID-19 patients in clinical trials was intravenous, and treatment dosage was nine times higher than its usual dosage for heartburn. The results of the initial clinical trial were crude and not statistically significant; thus further *in vitro* studies and clinical trials with a large group of COVID-19 patients need to be validated. Researchers are still not at a stage where they can tell conclusively whether this drug is effective and thus further research is needed, but its efficacy if proven could be a game-changer for treating COVID-19, as this medicine is low cost and there is also a good stock available.^92^

**Fig. 6:**
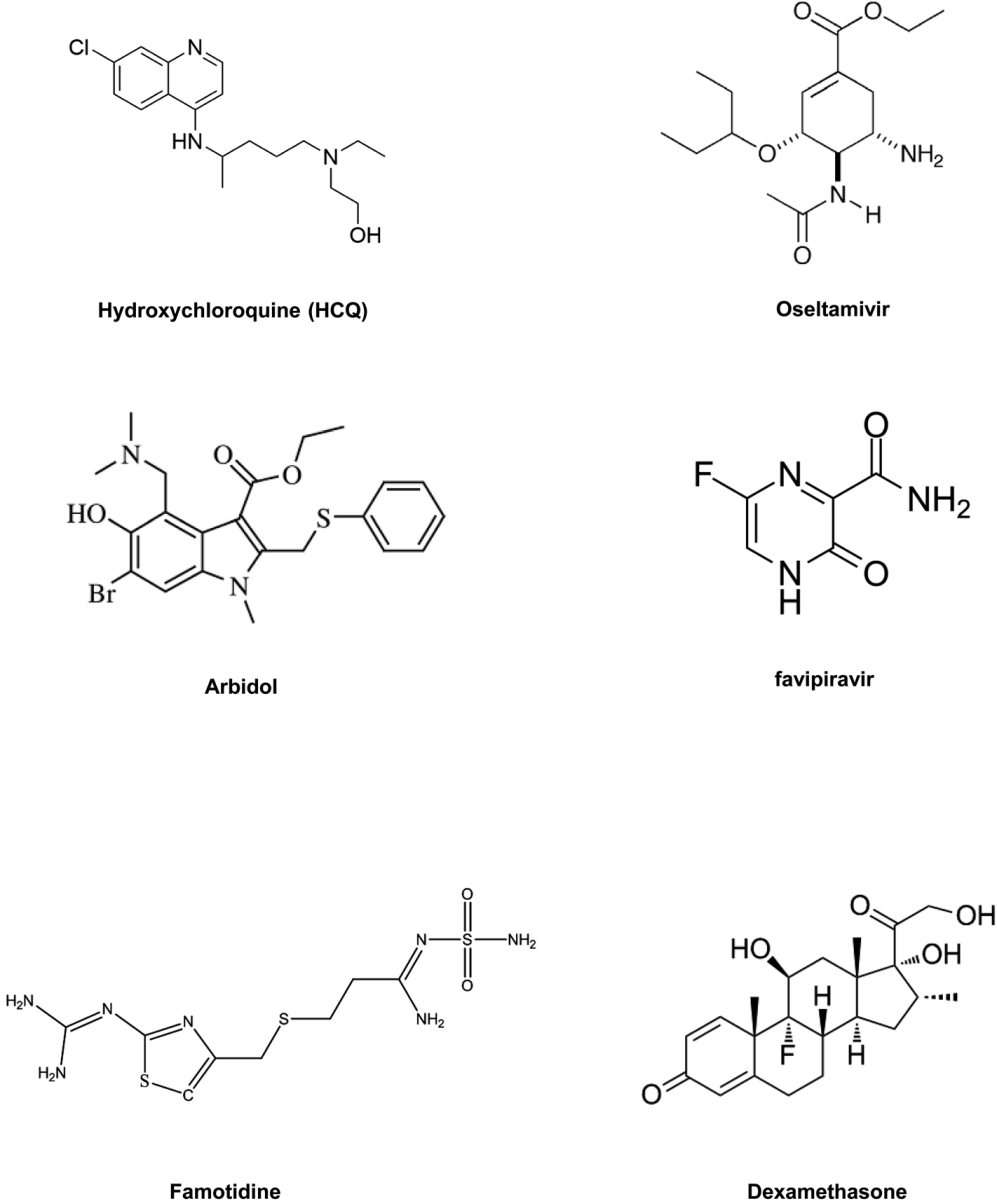
Repurposed small-molecule drugs.

In summary, drug repositioning is one of the best possible alternatives to solve this global health crisis. So, in this section, we have highlighted some of the broad range of promising antiviral drugs (Table 2) and their proposed mechanism of action and evaluated their efficacy against COVID-19.^84^Fig. 6 includes structures of the repurposed drugs discussed in the section on drug repurposing.

**Table 2:** Therapeutics for COVID-19 based on existing drugs

Drug candidate	Target	Classification	Mechanism of action	Reference
Chloroquine and hydroxychloroquine	Endosome-ACE2	Antimalarial drug	Prevent viral entry *via* inhibiting viral protein glycosylation and increasing endosomal pH	86, NCT01067417
Remdesivir, favipiravir, ribavirin, galidesiver	RdRp	Ebola, hepatitis C, Marburg virus	Nucleoside analog enters into the viral RNA chain, causing premature termination	81, NCT04257656, NCT04319900
Arbidol	Spike protein-ACE2	Influenza antiviral drug	Prevents viral cell fusion with host cells	91, NCT04260594, NCT04255017, NCT04252885
Oseltamivir	Exocytosis of new virus from host cells	Influenza antiviral drug	Prevents the secretion of neuraminidase from virus cells, which initiates the release of new virus into host cells	89, NCT04303299
Famotidine	PLpro	Antacid	Inhibits viral PLpro activity	92

## Monoclonal antibody based therapy

We have already discussed that RBD is an attractive target for neutralizing the monoclonal antibodies. In fact, it has been noted that most of the antibodies developed (about 90%) are specifically targeted on the S-protein which is the RBD. Monoclonal antibodies (mAbs) are undoubtedly one of the best candidates due to their extraordinary specificity towards the antigenic epitope and their possible dosage at an effective and consistent concentration, unlike plasma therapy. Monoclonal antibodies are not an alien entity in the pharmaceutical industry, considering that a large number of them are being marketed and used in treating disorders like rheumatoid arthritis^93^ and cancer,^94^ so the idea of identification, cloning and production of mAbs that will specifically target the spike protein of the virus to prevent its access into the host cells is going to be an attractive method in the prevention and treatment of COVID-19.

In one such study with 26 patients who have recovered from COVID-19, SARS-CoV-2 S1-targeted antibodies were found in large amounts, but when ELISA inhibition assay was performed to check the efficiency of these antibodies in blocking the interaction between SARS-CoV-2 RBD and hACE2, only 3 of the antibodies out of the 26 collected were successful.^95^ This observation is extremely important, as we learn from it that though all COVID-19 convalescent patients can produce anti-S1 as well as anti-RBD antibodies, in reality only a fraction of them can actually block the hACE2–RBD interaction.

Many pharmaceutical companies like AstraZeneca, Regeneron and Celltrion have already ramped up efforts to produce monoclonal antibodies that mostly targeted on the spike protein and may quickly emerge as some of the key players in this COVID-19 war.

AstraZeneca for instance, a British–Swedish pharmaceutical company, has partnered with academic institutions and governmental organizations to obtain support in preclinical testing so that they can fast-track their efforts to produce effective monoclonal antibodies.^96^

Celltrion, a biopharmaceutical company in South Korea, in partnership with the Korea Centers for Disease Control and Prevention (KCDC), secured 300 mAbs that bind to the spike protein of SARS-CoV-2. On screening them for their ability to neutralize the interaction between the spike protein in SARS-CoV-2 and hACE2 expressing Vero cells, 14 of the most potent antibodies have been extracted from the pool of 300 tested. Next up, Celltrion plans to carry out toxicity tests in mice before rolling out mass-scale production of the therapeutic mAbs in the market.^97^

Regeneron, an American biotechnology company, is developing a cocktail of antibodies instead of one. Their logic behind the development of the cocktail is the high mutation rates of viruses that will soon render the antibody ineffective. But by using a cocktail with a second or a third antibody, even when the first one loses its effectiveness the second or the third one in the cocktail will still be able to counter the virus.^98^

**Fig. 7:**
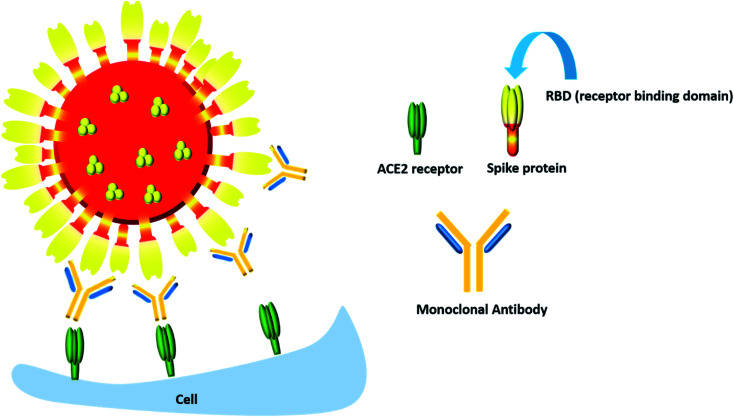
The monoclonal antibody mediated neutralization of the RBD of the spike protein of the COVID-19 virus.

Though most monoclonal antibodies are designed to inhibit the spike protein, one cannot deny that COVID-19 is also characterized by a “cytokine storm” which is accompanied by increased secretion of many pro-inflammatory cytokines, most notably IL-6 and IL-1β along with IL-17, tumor necrosis factor α (TNFα), G-CSF, IL-8, IP-10, CCL3, GM-CSF and MCP-1.^99^ Hence monoclonal antibodies which can dampen the effect of this inflammatory response can also be considered to be an important therapeutic component in the COVID-19 battle (Fig. 7).

One such clinical trial conducted in Anhui, China with mAb tocilizumab which targets IL-6 receptor (IL-6R), saw an immediate improvement in respiration and alleviation of fever in 21 COVID-19 patients with severe infection. Though more extensive randomized controlled trials are required to prove the efficacy of tocilizumab in COVID-19 patients with acute respiratory distress syndrome (ARDS), this should encourage more such clinical trials with other proinflammatory cytokines, like IL-1 and IL-17 receptors.^100^

Even though this antibody-based approach is a very powerful tool, monoclonal antibodies have their own share of shortcomings and pitfalls. Few studies with COVID-19 patients have reported an increase in the response of IgG and an extremely high titer of antibodies, which only suggested a bad prognosis in patients with an antibody-dependent enhancement (ADE) in the infections. A stimulation in the wrong direction can result in an adverse response in COVID-19 patients, leading to an enhancement in viral uptake mediated by antibodies and an intense inflammatory response. What is more worrisome is that a previous study with Middle East Respiratory Syndrome (MERS) virus has shown that a neutralizing mAb that targeted the spike protein in MERS eventually led to an antibody-dependent entry of the virus into the host cells.^101^ If such a pathogenic impact is found in mAbs developed against SARS-CoV-2, then the results will be devastating. To avoid such confusion in future, large-cohort studies should be conducted in order to either dismiss this theory or to substantiate the claim.

## Convalescent plasma therapy

**Fig. 8:**
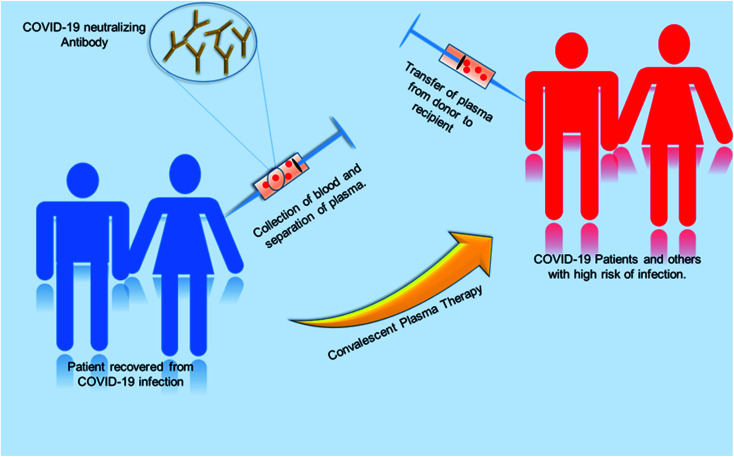
A schematic diagram representing the workflow for convalescent plasma therapy.

Globally, various initiatives have been taken to develop effective therapies in a fast-track mode. One of the promising approaches is convalescent plasma therapy. Plasma is the yellowish liquid component of blood that holds the blood cells in a whole blood suspension. Convalescent plasma means plasma that comes from people who have recovered from an infection, like SARS-CoV-2.^102^ From experience from the prior outbreak of SARS-CoV-1, it was known that such convalescent plasma contains neutralizing antibodies to the relevant virus. A study using convalescent plasma from 87 positive patients shows that the lowest level of anti-SARS neutralizing antibody titer detected by the neutralization assay was 1 : 12 and the highest titer was 1 : 512. The geometric mean of this convalescent plasma was 1 : 61. Test results show the stability of SARS specific neutralizing antibody level and the antibody level persisted as long as 180 days with a slight decline from 1 : 67 to 1 : 40 after 121 days after the onset of symptoms.^103^ The convalescent plasma therapy involves the separation of plasma from the whole blood of recovered patients through apheresis (a procedure called plasmapheresis) and to use the plasma for either prophylaxis of infection or treatment of disease.^102,104^ The theory behind the plasma therapy is that when a recovered patient's antibody (present in plasma) specific to a particular disease is ingested into someone under treatment, the antibody will specifically target the pathogen and help in recovery of the second patient. This therapy is akin to passive immunization. Passive antibodies work either by neutralizing the pathogen or by modifying the inflammatory response, which can be achieved during early stage of the disease.^104,115^ A meta-analysis showed a statistically significant 75% reduction in the odds of mortality and viral load among patients with severe acute respiratory infections (SARI) caused by a related corona virus and treated with convalescent plasma.^105^ SARS patient treated with ribavirin, high-dose steroid and convalescent plasma had better recovery rate then with ribavirin, high-dose steroid and continuing high-dose methylprednisolone.^107^ Patients who were given convalescent plasma before the 14th day of disease had a high rate of day-22 discharge rate and early recovery. So from this it can be inferred that this therapy is more useful if it is administered in the early stage of the disease.^108^ Convalescent plasma treatment was effective for patients infected by pandemic influenza A (H1N1) 2009.^112^ Many other studies were done on SARS and MERS infected patients, which suggest convalescent plasma treatment can reduce the disease state and provide a good clinical outcome.^113,114^ The previous knowledge and success story had directed treatment using plasma therapy for COVID-19 infected patients. Various studies with COVID-19 patients showed promising results.^104,106,109–111^Fig. 8 shows the procedure of plasma therapy from donor to recipient patients and health workers.

However, studies so far have been performed with a small number of patients; therefore to make conclusions about the success of this therapy against COVID-19, we need to have more data for patients who have undergone plasma therapy trials.

## Interferon based therapy

Type I interferons (IFN-I) are produced by the body as the first line of defense against the virus. Type I interferons are cytokines consisting of subtypes α, β, κ and ε.^116^ They all bind to interferon alpha receptors (IFNAR) consisting of chains IFNAR 1 and 2. They are produced by a variety of cells, including macrophages and NK cells, but their most potent source of production are plasmacytoid dendritic cells, which possess special pattern recognition receptors (PRR) to sense the viral components as a signal to immediately start secretion of IFN-I.^117^

As IFN-I binds on IFNAR, it induces phosphorylation of STAT1 (Signal Transducer and Activators of Transcription 1), which then localizes to the nucleus, activating interferon-stimulated genes (ISGs). ISGs activate a variety of antiviral proteins and inflammatory signaling molecules, which include RIG-I (retinoic acid-inducible gene), Protein Kinase R (PKR) and Mx, which together help to detect virus particles, inhibit the viral replication process, and prevent viral coat assembly and the release of newer viral progeny. IFN-I can therefore be deemed important in antiviral therapy without an iota of doubt.^118^

IFN-I treatment has been studied in both pre-existing coronaviruses in both animal and cellular models in combination with other antiviral drugs ritonavir and remdesivir.^118^ Though IFN α and β indicated success in both cellular and animal models,^120^ they did not work significantly well in human beings.^121^ Though one thing has been particularly noted: that improvement was observed only in patients who did not suffer from comorbidities.^122^

One of the possible reasons for the failure of IFN-I treatment against SARS-CoV may be due to its higher defense system against interferon I, but on the positive side is that SARS-CoV-2 is less capable of suppressing IFN production as sequence analysis revealed that the amino acid composition of these IFN-I antagonistic proteins is quite different in SARS-CoV-2 compared to SARS-CoV and may not interfere with the interferon responses as aggressively as observed in SARS-CoV.^123^ Also SARS-CoV-2 does not express two major IFN-I inhibitor proteins of SARS-CoV, namely ORF3b and ORF6. ORF3b blocks phosphorylation of IRF3, which is a major protein involved in activating the IFN response and ORF6 plays a major role in blocking the production of IFN-I by disrupting the transport of karyopherin and inhibition of JAK-STAT1 mediated signaling in the case of SARS-CoV.^124,125^

Between IFN α and β, IFN β has shown more potent activity against coronaviruses.^126^ In a series of studies conducted with IFNβ1b or IFNβ1a, both were found to possess inhibitory activity against SARS-CoV and MERS-CoV. Hence IFNβ1 seems to be the likely candidate for studies with SARS-CoV-2. In fact, clinical studies with IFNβ1a saw an improvement in the acute respiratory distress syndrome (ARDS) condition in patients with reduced vascular leakage.^127^ But this activity of IFN-I is only applicable if the patients are given treatment shortly after the infection. When treatment was provided at later stages of the infection, IFN-I failed to ameliorate ARDS conditions.^128^

A recent study has shown that spraying of IFNα2b can be used as a prophylactic agent.^123^ In another study conducted among 2000 susceptible healthcare workers in Hubei, it was observed that recombinant IFN-α (rhIFN-α) nasal drops administered with or without thymosin α1 to low and high risk individuals respectively over a period of 28 days protected all of them from catching COVID-19 pneumonia.^129^ Hence the two studies prove that IFN-1 can be used in healthy and susceptible individuals as a means to prevent SARS-CoV-2 associated infection. No such prophylactic activity of IFN-I has been observed in SARS-CoV or MERS-CoV.^119,130^

As we are all aware of the cytokine storm associated with SARS-CoV-2 infections, many reports have emerged claiming that the pulmonary lesions observed in COVID-19 infections may be an excessive IFN-I response, which leads to tearing and shearing of the pulmonary tissues. If this is to be believed, then the IFN-I based treatments have to be limited to the early phases of COVID-19 infection as in the later phases they might be fatal.^131^ In fact, in the later phases of infection, anti-interferon based therapeutics might actually do wonders.^132^

In China a combination of IFN-I is being administered with lopinavir/ritonavir, ribavirin or remdesivir, which could increase the efficiency of treatment against COVID-19.^119^ In a recent study with recombinant human interferons (IFNα/β) conducted in Vero cells infected with SARS-CoV-2, it has been reported that it reduces the viral titers significantly.^133^ This means that an exogenous treatment with IFN-β actually further enhances the interferon-mediated antiviral response, which helps in a speedy recovery.

Apart from interferon beta, even interferon alpha might emerge as one of the leading treatment strategies. In one such study conducted with 77 patients in Wuhan, those who were treated with interferon alpha-2b (IFNα-2b) had a shorter duration of virus shedding and even showed a drop in the levels of inflammation-inducing IL-6 cytokine.^134^ This means if a more stable molecule like a pegylated version of IFNα-2b is created, it will not only reduce the overall viral load but also cause a reduction in the levels of IL-6 cytokine. Interferon alpha-2b has already been approved in countries like China and Cuba for the treatment of COVID-19 patients and a leading pharmaceutical company named Zydus Cadila has sought permission from regulatory agencies to take its pegylated form of IFNα-2b to clinical trials.

IFN lambdas or type III interferons do not share a high homology with the other families of interferons, but what would be significant to mention here is their ability to maintain a stable antiviral response in the pulmonary tract, which subdues any chances of a cytokine storm. Their chief advantage over IFN-I is that they are induced when the viral load is still low and therefore can immediately impede viral replication, naturally bringing the viral load down much earlier than type I IFNs.^135^ Along with stopping the hyper-inflammatory cytokine response, IFN λ also induces an adaptive immune response by activation of the cyto-toxic T lymphocytes (CTLs), Th1 (T-helper cell) responses and B-lymphocyte driven humoral responses that are pivotal for maintaining immunity for a longer duration.^136^ So, it is quite clear that IFN λ acts alongside type I IFNs to fine tune the clearance of viral load along with minimal damage due to the inflammatory response of the cytokines.^135^

Along with most pros, there are still a few questions regarding the use of IFN λ in the COVID-19 fight whose one pegylated form (peg-IFN λ1) is already available and has been used successfully in clinical trials against chronic hepatitis D virus. Some of the important questions that can be raised regarding the usage of IFN λ are whether their receptor IFNLR1 could have a higher expression in an inflammation-prone environment in the lungs, thereby increasing the adverse effects that could be caused by IFN λ on cells in human beings; whether the immune cells respond at all to IFN λ during a COVID-19 infection as they are known to impede inflammatory effects; and whether the low expression pattern of IFNLR1, however advantageous in evading the hyper-inflammatory responses it might be, does not restrict the efficacy of the treatment. Whatever the issue, the need for good prophylactic agents in order to induce an antiviral state and prevent the development of ARDS in COVID-19, high hopes have been pinned on the therapeutic potential of IFN λ along with the other IFNs like IFN-I.

## Development of a vaccine for the management of COVID-19

### DNA-based vaccine

Vaccination is the process of administering a molecule or combination of molecules that stimulates the active acquired immunity of a host against the infectious agent in such a manner that no harm is caused to the host. A new radical approach in vaccination is a DNA vaccine where a plasmid containing a DNA sequence encoding the antigen is inserted into the host cells, which leads to subsequent expression of its products in the host cells and induces both cellular and humoral immunity against the antigen, thus, protecting host cells from the infection.

In 2004 Yang, Z. *et al.* used the same DNA vaccine technology to immunize mice with an expression vector encoding the spike (S) glycoprotein of the SARS-CoV Urbani strain.^137^ As a result, the mouse model showed an increase in S glycoprotein specific CD4^+^ T-cell immune response and protective humoral immunity. This humoral immunity can help to prevent the replication of the virus in the mouse model. Due to the high similarity (80%) between SARS-CoV-2 and other human coronaviruses (SARS-like bat CoV)^138^ the approach they used to develop a vaccine can also be used against SARS-CoV-2 in the full length or a short segment of the spike protein. A pharma company called Inovio has already started its clinical phase trial 1 for DNA vaccine INO-4800 against SARS-Cov-2.^139^

### mRNA-based vaccine

DNA and the mRNA vaccine are ideal technologies and a possible alternative to a traditional vaccine to fight pandemics like COVID-19. This shows a need to be explicit about precisely what is meant by the word ‘mRNA vaccine’. mRNAs as a vaccine platform were first promoted in 1991. Cells use DNA as a starting material for protein production through an RNA intermediate, where mRNA is used as a template material. Similarly, an RNA vaccine consists of specially designed strands of mRNA that code for a disease-specific antigen. Once these RNAs have entered into the host cells, the cellular translational machinery produces a fully functional antigenic protein from this mRNA. Subsequently, this antigenic protein is taken up by the antigen-presenting cells to activate the innate immune response, CD4^+^, and CD8^+^ cells.^140^

Naked mRNAs are susceptible to degradation by extracellular RNases. Thus, several transfecting agents have been developed to facilitate the cellular uptake of mRNA and to protect against its degradation, such as viral vectors, nonviral methods (gene gun, electroporation), lipids, and biomaterial based nanoparticles.^141^ For infectious diseases (rabies, HIV, and Zika virus)^142–144^ mRNA vaccines are being investigated as a way to produce vaccines more rapidly, particularly in response to an emerging outbreak like COVID-19. In the above context, the mRNA vaccine mRNA-1273 was developed by scientists from the National Institute of Allergy and Infectious Diseases (NIAID) and their collaborators, which is a new type of lipid nanoparticle encoded mRNA encoding S protein.^145^ The vaccine has shown promising results in clinical trials and was already assigned for phase 1 clinical trials in March 2020.^146^ However, the full effect of the vaccine on humans still needs to be validated.

### Virus like particle vaccine

Virus like particles (VLPs) are a specific class of subunit vaccine where antigenic proteins of the virus are expressed in various expression vectors and then assembled into a structure that mimics the live virus structurally.^147^ VLPs are non-infectious as they lack the genetic material (DNA or RNA), but VLPs have a similar extent of antigenicity to a live virus. Depending on the complexity of a VLP, it can express one or several viral structural proteins.^148^

VLPs have been shown to elicit a B cell mediated immune response as well as cytotoxic T lymphocytes. Therefore, this role of VLPs was one of the significant factors for choosing them as a potential vaccine candidate, especially for infectious diseases like MERS-CoV, SARS-CoV and SARS-CoV-2, which cause high morbidity and death.^149^ In 2018 scientists generated VLPs of MERS-CoV by co-expressing S (spike), E (envelope), M (membrane) genes in a baculovirus expression system.^149^ The VLPs were able to generate virus-neutralizing antibodies in rhesus macaques.^150^ This study shows promising results for using VLPs as a potential vaccine against SARS-CoV-2.

Novavax in February 2020 announced that it has started animal trials for its vaccine based on antigens derived from the spike (S) protein of SARS-CoV-2 using novel proprietary recombinant protein nanoparticle platform; it is much like a VLP.^151^

### Protein-based vaccine

Protein-based vaccines are a type of subunit vaccine where the component or antigenic part of a pathogen that is best suited for stimulation of the immune response is chosen to immunize a host to develop a specific immune response against that pathogen.^152^ They differ from inactivated or live attenuated vaccines since they contain only the antigenic part of the pathogen not the whole pathogen itself.^153^

In 2006 Kam, Y. W. *et al.* produced a vaccine for SARS-CoV based on recombinant native full-length spike protein trimers (triSpike). The trispike was capable of eliciting an immune response *in vivo* and provided a course of action for a human vaccine.^154^

In April 2020 Sanofi, a French multinational pharmaceuticals company, announced a collaboration with GlaxoSmithKline (GSK), a British pharmaceuticals company, to fight COVID-19. The technology will use the vaccine technology of a flu virus developed by Sanofi along with GSK's AS03 adjuvant.^155^

### Live attenuated vaccine

Live attenuated vaccines use the “wild type” of diseases causing pathogens. However, these wild pathogens or viruses do not cause any disease since they are weakened under laboratory conditions by different methods like serial dilution, codon deoptimization, or growing the pathogen in multiple hosts to induce mutations.^156^ The weakened virus replicates in a host cell. It stimulates a robust immune response, and the immune response to that of a live attenuated vaccine is identical to natural infection by a wild type of pathogen.^157^

Menachery, V. D. *et al.* showed a possible target for the attenuation of coronavirus. They manipulated NSP16, a conserved 2′-*O*-methyltransferase (MTase) paired with other attenuated approaches to develop a live attenuated strain to be further used for vaccine development.^158^ Similarly, a SARS-CoV-2 vaccine could be acquired through a live attenuated strain and later infecting the person with the attenuated strain to develop a robust immune response. Recently, in 2020 Codagenix has already announced a collaboration with the serum institute of India to develop a live attenuated vaccine for SARS-Cov-2.^159^

### Inactivated or killed antigen vaccine

A vaccine of this type is created by killing the pathogen through some physical (UV light)^160^ or chemical (β-propiolactone, formaldehyde, sodium hypochlorite)^161^ agents. This killed pathogen has lost the ability to cause disease but retains its capacity to develop an immune response in the host. Since they are killed or inactivated strains of the pathogen, they cannot revert to their pathogenic form. However, when infected, they do protect against the real pathogen.

Several studies have been done on inactivated SARS-CoV^162^ and MERS^163^ vaccines. The outcomes of these studies provide an opportunity to develop a vaccine candidate in a similar way for the SARS-CoV-2 outbreak. Gao, Q. *et al.* in their recent article, described a method for the rapid development of vaccines using inactivated SARS-CoV-2, where they collected different strains of SARS-Cov-2 from COVID-19 infected patients.^164^ Among them the CN2 strain was chosen to develop a vaccine, while other strains were used as a preclinical challenge strain. Purified inactivated SARS-CoV-2 virus vaccine candidate (PiCoVacc) was used to infect mice, rats, and non-human primates, which induced SARS-CoV-2 specific neutralizing antibodies. This data supports the potential vaccine candidate for SARS-CoV-2.

**Fig. 9:**
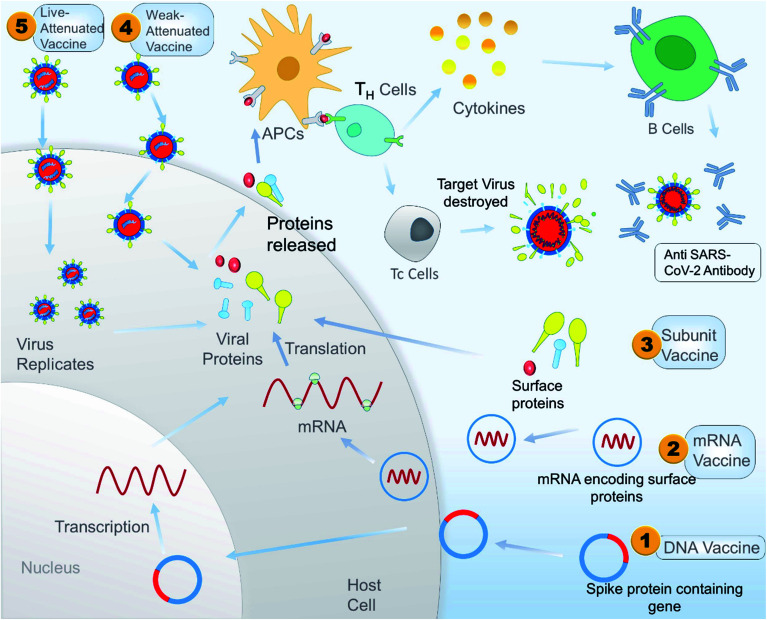
A schematic illustrated mechanism of different types of potential vaccine for SARS-CoV-2 in the context of developing immunity in the host.

Antibody dependent enhancement (ADE) is a disadvantage associated with plasma therapy as well as with different vaccine treatments. A study showed that inactivated SARS-CoV vaccine can induce ADE in rhesus macaques. The study also showed that antibodies against spike glycoproteins S471–503, S604–625, and S1164–1191 can efficiently prevent infection in non-human primates, but antibody targeting peptides S597–603 can enhance infection by ADE. So during the design of a peptide-based vaccine, peptides S597–603 need to be eliminated to avoid ADE. A peptide-based vaccine could be a better alternative than a whole protein vaccine because it gives us an option to identify and eliminate the epitope sequence that is responsible for ADE.^165^ A schematic illustration of the overall mechanism of action of different vaccines is shown in Fig. 9, and Table 3 constitutes a list of the vaccines under clinical trials as per WHO on 15 May 2020.

**Table 3:** A list of under-development vaccine candidates for COVID-19, according to the WHO as of 15 May 2020. Ref. – Draft Landscape of COVID 19 candidate vaccines, https://www.who.int/who-documents-detail/draft-landscape-of-covid-19-candidate-vaccines, accessed on May 15, 2020^a^

Vaccine platform/type of candidate	Immunogen/target	Current stage/phase of trial	Advantages	Disadvantages	Reference
RNA-based (mRNA-1273)	LNP-encapsulated mRNA VACCINE encoding the S protein	Phase 1 (NCT04283461), phase 1 (2020-001038-36), phase 1/phase 2 (NCT04368728)	Less infectious or mutagenic, short life span, chance of integration into the host genome is minimum, a low dose is required	Vaccines need optimal delivery agents, safety issues with unintended immune responses	166 and 167
Viral vector	Attenuated adenovirus capable of producing the spike (S) protein of SARS-CoV-2, ChAdOx1 (chimpanzee adenovirus vaccine vector)	Phase 2 (ChiCTR2000031781), phase 1 (ChiCTR2000030906), phase 1/phase 2 (NCT04324606)	High immunogenicity, long-term expression of infectious genes	Risk of pathogenesis and tumorigenesis	168
Subunit	Short antigenic peptide sequence	Pre-clinical AJ Vaccines, Epivax, Novavax, GSK/Sanofi	Induce both cellular and humoral immunity, chance of inducing diseases or side-effects is minimum	Multiple boosters required, an adjuvant is needed for delivery	169
Virus-like particles (VLPs)	Plant derived VLPs that mimic the shape and dimensions of the virus	Pre-clinical Medicago, Adaptvac	Can develop immunity against a multimeric protein at a given time	Assembly of VLPs in an expression vector is intricate	170 and 171
Inactivated	Inactivated SARS-CoV-2 + alum, inactivated SARS-CoV-2	Phase 1 (NCT04352608), Phase 1 (ChiCTR2000031809, ChiCTR2000032459)	Much safer as compared to a live attenuated vaccine, has been tested in the case of SARS-CoV-1	First dose does not always induce a robust immune response, antigenic integrity needs to be maintained	172 and 173
Live-attenuated virus	Whole virion	Phase 3 (NCT04328441), (NCT0432726) [BRACE] Pre-clinical Codagenix/Serum Institute of India	Memory cells are generated, producing the same effect as that of a live infectious pathogen	Might replicate in an uncontrolled manner, safety concerns	174 and 175
DNA-based (INO-4800)	DNA plasmid encoding the S protein	Phase 1 (NCT04336410)	Multiple variants of antigen can be inserted into a single plasmid	Chance of incorporation into the host genome, activation of oncogenes	176 and 177

a BRACE: BCG vaccination to protect healthcare workers against COVID-19.

## Complementary defenses by natural products and herbal medicine

Natural products and herbal products or medicines have shown potential in a plethora of diseases, including viral infection, from ancient times to the modern age. They are relatively safe and often possess multifactorial benefits. In the context of COVID-19, there has been usage of the antimalarial drug chloroquine phosphate, a derivative of quinine, which is extracted from the bark of cinchona trees.

Recently, Shaanker, R. *et al.* showed that a combination of natural products from commonly used spices, fruits and vegetables, some of which are used in the cuisine in India and other countries (apigenin, coriandrin, curcumin, glabridin, glycoumarin, glycyrrhizin, hederagenin, liquiritigenin, oleanolic acid, quercetin, rosmarinic acid, safficinolide, sageone, ursolic acid, glucobrassicin) has potential against COVID-19 6LU7 and 6Y2E protease.^178^ Furthermore, Chen, L. *et al.* suggested that nine phytochemicals (amaranthine, methyl rosmarinate, 5,7,3′,4′-tetrahydroxy-2′-(3,3dimethylallyl)isoflavone myricitrin, 3,5,7,3′,4′,5′-hexahydroxy flavanone-3-*O*-beta-d glucopyranoside, (2*S*)-eriodictyol 7-*O*-(6′′-*O*-galloyl)-beta-d-glucopyranoside, calceolarioside B, myricetin 3-*O*-beta-d glucopyranoside, licoleafol) from different plant sources may have an inhibitory effect on SARS COV-2 3CLpro activity as well as virus replication. Licorice, *Glycyrrhiza glabra*, a very important herb in China, with many pharmacological activities has been used to control COVID-19 symptoms.^179,180^ Based on recent studies, currently in the preprint stage, a group of researchers reported that *Withania somnifera* might prove to be an essential medicine in combating COVID-19 by disrupting interactions between viral S protein RBD and host ACE2 receptor.^181^ In another recent development, also in the preprint stage, a group of researchers has analyzed the potency of this herbal medicine as a COVID-19 warrior by suggesting that *Withania somnifera* targets Mpro viral enzyme, which plays a vital role in the replication and spread of the virus.^182^

Apart from all the therapeutic options, the benefits of herbal medicine for treating multiple diseases in humans have been considered for ages and cannot be ignored. Herbal medicines also boost the human immune system, which may help to combat the spread of the virus and reduce the fatal power of the virus.

## Conclusions

Undoubtedly this massive outbreak of COVID-19 has been a major issue for humankind since its emergence, but around the globe, the scientific community is also trying its best to overcome its deadly effects. Amidst the lockdown and emergency period of this pandemic, the rapid continuation of scientific research for the development of vaccines or drugs is itself quite challenging. Moreover, a major barrier to this is the very short span of time within which research results are generated, along with corresponding age and geographical location biased data and limited sample sizes that can be used for clinical trials. These shortcomings not only increase the risk of an unsuccessful clinical translation to the mass population but can also influence viral mutation towards a more deadly strain. Despite several efforts and trials, any production of a possible vaccine is still a long way off, and in this intermediate period, therapeutics which were previously successful in defying SARS-CoV and MERS-CoV are being used to defeat this novel viral strain to some extent. But in this regard, a proper authenticated database of clinical trial results with repurposed drugs is still not available. Thus, drug repurposing studies are being altered and affected. Based on recent reports, HCQ is still a debatable drug for consideration for COVID-19 treatment. Thus further, more randomized clinical trials are required to validate the results, and, in this respect, the WHO has lifted the ban on the use of HCQ in a solidarity trial, which was previously imposed for a few days.^183^ Favirapir and remdesivir are now gaining much attention among repurposed drugs. Recently, dexamethasone, a type of corticosteroid medication used in other diseases to reduce inflammation, has also emerged as a potential COVID-19 life-saving drug in initial clinical trials.^184^ But, before making any conclusive statement about the efficacy of this repurposed drug for COVID-19 treatment, more randomized clinical trials and other scientific validation need to be performed.

## Conflicts of interest

The authors declare no competing financial interests.

## Supplementary Material

## References

[cit1] Chen Y., Liu Q., Guo D. (2020). J. Med. Virol..

[cit2] Fehr A. R., Perlman S. (2015). Methods Mol. Biol..

[cit3] Wenzhong L., Hualan L. (2020). ChemRxiv.

[cit4] Zhao J., Yang Y., Huang H. P. (2020). et al.. medRxiv.

[cit5] Understanding the risky combination of diabetes and the coronavirus by American HeartAssociationNews, https://www.heart.org/en/news/2020/04/13/understanding-the-risky-combination-of-diabetes-and-the-coronavirus, accessed on May 20,2020

[cit6] Stower H. (2020). Nat. Med..

[cit7] Cao B., Wang Y., Wen D., Liu W., Wang J., Fan G., Ruan L., Song B., Cai Y., Wei M., Li X. (2020). et al.. N. Engl. J. Med..

[cit8] Meo S. A., Klonoff D. C., Akram J. (2020). Eur. Rev. Med. Pharmacol. Sci..

[cit9] Shanmugaraj B., Siriwattananon K., Wangkanont K., Phoolcharoen W. (2020). Asian Pac. J. Allergy Immunol..

[cit10] Rockx B., Kuiken T., Herfst S., Bestebroer T., Lamers M. M., Munnink B. B. O., de Meulder D., van Amerongen G., van den Brand J., Okba N. M., Schipper D. (2020). Science.

[cit11] Lu R., Zhao X., Li J., Niu P., Niu B., Yang H., Wu W., Wang W., Song H., Huang B., Zhu N., Bi Y. (2020). Lancet.

[cit12] Walls A. C., Tortorici M. A., Snijder J., Xiong X., Bosch B. J., Rey F. A., Veesler D. (2017). PNAS.

[cit13] Jia H. P., Look D. C., Shi L., Hickey M., Pewe L., Netland J., Farzan M., Wohlford-Lenane C., Perlman S., McCray P. B. (2005). J. Virol..

[cit14] Yeo C., Kaushal S., Yeo D. (2020). Lancet Gastroenterol. Hepatol..

[cit15] Das G., Mukherjee N., Ghosh S. (2020). ACS Chem. Neurosci..

[cit16] Letko M., Marzi A., Munster V. (2020). Nat. Microbiol..

[cit17] Yan R., Zhang Y., Li Y., Xia L., Guo Y., Zhou Q. (2020). Science.

[cit18] Wan Y., Shang J., Graham R., Baric R. S., Li F. (2020). J. Virol..

[cit19] Liu W. R., Morse J. S., Lalonde T., Xu S. (2020). ChemBioChem.

[cit20] Adedeji A. O., Severson W., Jonsson C., Singh K., Weiss S. R., Sarafianosa S. G. (2013). J. Virol..

[cit21] Kao R. Y., Tsui W. H., Lee T. S., Tanner J. A., Watt R. M., Huang J. D., Hu L., Chen G., Chen Z., Zhang L., He T. (2004). Chem. Biol..

[cit22] Li W., Moore M. J., Vasilieva N., Sui J., Wong S. K., Berne M. A., Somasundaran M., Sullivan J. L., Luzuriaga K., Greenough T. C., Choe H. (2003). Nature.

[cit23] Matsuyama S., Nagata N., Shirato K., Kawase M., Takeda M., Taguchi F. (2010). J. Virol..

[cit24] Hoffmann M., Kleine-Weber H., Schroeder S., Krüger N., Herrler T., Erichsen S., Schiergens T. S., Herrler G., Wu N. H., Nitsche A., Müller M. A. (2020). Cell.

[cit25] Kawase M., Shirato K., van der Hoek L., Taguchi F., Matsuyama S. (2012). J. Virol..

[cit26] Zhou Y., Vedantham P., Lu K., Agudelo J., Carrion Jr R., Nunneley J. W., Barnard D., Pöhlmann S., McKerrow J. H., Renslo A. R., Simmons G. (2015). Antiviral Res..

[cit27] Zhang J., Ma X., Yu F., Liu J., Zou F., Pan T., Zhang H. (2020). bioRxiv.

[cit28] Coutard B., Valle C., de Lamballerie X., Canard B., Seidah N. G., Decroly E. (2020). Antiviral Res..

[cit29] Bosch B. J., Bartelink W., Rottier P. J. M. (2008). J. Virol..

[cit30] Matsuyama S., Ujike M., Morikawa S., Tashiro M., Taguchi F. (2005). Proc. Natl. Acad. Sci. U. S. A..

[cit31] Andersen K. G., Rambaut A., Lipkin W. I., Holmes E. C., Garry R. F. (2020). Nat. Med..

[cit32] Garten W., Hallenberger S., Ortmann D., Schäfer W., Vey M., Angliker H., Shaw E., Klenk H. D. (1994). Biochimie.

[cit33] Millet J. K., Whittaker G. R. (2014). Proc. Natl. Acad. Sci. U. S. A..

[cit34] Zhu N., Zhang D., Wang W., Li X., Yang B., Song J., Zhao X., Huang B., Shi W., Lu R., Niu P. (2020). N. Engl. J. Med..

[cit35] Wu F., Zhao S., Yu B., Chen Y. M., Wang W., Song Z. G., Hu Y., Tao Z. W., Tian J. H., Pei Y. Y., Yuan M. L. (2020). Nature.

[cit36] Zhou P., Yang X. L., Wang X. G., Hu B., Zhang L., Zhang W., Si H. R., Zhu Y., Li B., Huang C. L., Chen H. D. (2020). Nature.

[cit37] Xu X., Chen P., Wang J., Feng J., Zhou H., Li X., Zhong W., Hao P. (2020). Sci. China: Life Sci..

[cit38] Qamar M. T. Ul., Alqahtani S. M., Alamri M. A., Chen L.-L. (2020). J. Pharm. Anal..

[cit39] Yang H., Xie W., Xue X., Yang K., Ma J., Liang W., Zhao Q., Zhou Z., Pei D., Ziebuhr J., Hilgenfeld R. (2005). PLoS Biol..

[cit40] Chu C. M., Cheng V. C. C., Hung I. F. N., Wong M. M. L., Chan K. H., Chan K. S., Kao R. Y. T., Poon L. L. M., Wong C. L. P., Guan Y., Peiris J. S. M. (2004). Thorax.

[cit41] de Wilde A. H., Jochmans D., Posthuma C. C., Zevenhoven-Dobbe J. C., van Nieuwkoop S., Bestebroer T. M., van den Hoogen B. G., Neyts J., Snijder E. J. (2014). Antimicrob. Agents Chemother..

[cit42] Cao B., Wang Y., Wen D., Liu W., Wang J., Fan G., Ruan L., Song B., Cai Y., Wei M., Li X., Xia J. (2020). N. Engl. J. Med..

[cit43] Xu Z., Peng C., Shi Y., Zhu Z., Mu K., Wang X., Zhu W. (2020). et al.. bioRxiv.

[cit44] Báez-Santos Y. M., St. John S. E., Mesecar A. D. (2015). Antiviral Res..

[cit45] Ratia K., Kilianski A., Baez-Santos Y. M., Baker S. C., Mesecar A. (2014). PLoS Pathog..

[cit46] Kiira R., Pegan S., Takayama J., Sleeman K., Coughlin M., Baliji S., Chaudhuri R., Fu W., Prabhakar B. S., Johnson M. E., Baker S. C., Ghosh A. K., Mesecar A. D. (2008). PNAS.

[cit47] Ghosh A. K., Takayama J., Rao K. V., Ratia K., Chaudhuri R., Mulhearn D. C., Lee H., Nichols D. B., Baliji S., Baker S. C., Johnson M. E. (2010). J. Med. Chem..

[cit48] Zhang L., Lin D., Sun X., Curth U., Drosten C., Sauerhering L., Becker S., Rox K., Hilgenfeld R. (2020). Science.

[cit49] Kirchdoerfer R. N., Ward A. B. (2019). Nat. Commun..

[cit50] Chiou H. E., Liu C. L., Buttrey M. J., Kuo H. P., Liu H. W., Kuo H. T., Lu Y. T. (2005). Chest.

[cit51] Stockman L. J., Bellamy R., Garner R. (2006). PLoS Med..

[cit52] Smith E. C., Blanc H., Vignuzzi M., Denison M. R. (2013). PLoS Pathog..

[cit53] Ferron F., Subissi L., De Morais A. T. S., Le N. T. T., Sevajol M., Gluais L., Decroly E., Vonrhein C., Bricogne G., Canard B., Imbert I. (2018). PNAS.

[cit54] Sheahan T. P., Sims A. C., Graham R. L., Menachery V. D., Gralinski L. E., Case J. B., Leist S. R., Pyrc K., Feng J. Y., Trantcheva I., Bannister R., Park Y., Babusis D., Clarke M. O., Mackman R. L., E Spahn J., Palmiotti C. A., Siegel D., Ray A. S., Cihlar T., Jordan R., Denison M. R., Baric R. S. (2017). Sci. Transl. Med..

[cit55] Agostini M. L., Andres E. L., Sims A. C., Graham R. L., Sheahan T. P., Lu X., Smith E. C., Case J. B., Feng J. Y., Jordan R., Ray A. S., Cihlar T., Siegel D., Mackman R. L., Clarke M. O., Baric R. S., Denison M. R. (2018). mBio.

[cit56] Wang M., Cao R., Zhang L., Yang X., Liu J., Xu M., Shi Z., Hu Z., Zhong W., Xiao G. (2020). Cell Res..

[cit57] Williamson B. N. , Feldmann F., Schwarz B., Meade-White K., Porter D. P., Schulz J., Van Doremalen N., Leighton I., Yinda C. K., Pérez-Pérez L. and Okumura A., bioRxiv, 2020, 10.1101/2020.04.15.043166

[cit58] Holshue M. L., DeBolt C., Lindquist S., Lofy K. H., Wiesman J., Bruce H., Spitters C., Ericson C., Wilkerson S., Tural A., Diaz G. (2020). N. Engl. J. Med..

[cit59] Chang Y. , Tung Y., Lee K., Chen T., Hsiao Y., Chang H., Hsieh T., Su C., Wang S., Yu J., Shih S., Lin Y., Lin Y., Tu Y. E., Tung C. and Chen C., 2020, Preprints, 10.20944/preprints202002.0242.v1

[cit60] Zumla A., Chan J. F., Azhar E. I., Hui D. S., Yuen K. Y. (2016). Nat. Rev. Drug Discovery.

[cit61] Tanner J. A., Zheng B. J., Zhou J., Watt R. M., Jiang J. Q., Wong K. L., Lin Y. P., Lu L. Y., He M. L., Kung H. F., Kesel A. J. (2005). Chem. Biol..

[cit62] Kim M. K., Yu M. S., Park H. R., Kim K. B., Lee C., Cho S. Y., Kang J., Yoon H., Kim D. E., Choo H., Jeong Y. J. (2011). Eur. J. Med. Chem..

[cit63] Adedeji A. O., Singh K., Calcaterra N. E., DeDiego M. L., Enjuanes L., Weiss S., Sarafianos S. G. (2012). Antimicrob. Agents Chemother..

[cit64] Adedeji A. O., Singh K., Kassim A., Coleman C. M., Elliott R., Weiss S. R., Frieman M. B., Sarafianos S. G. (2014). Antimicrob. Agents Chemother..

[cit65] Walls A. C., Park Y. J., Tortorici M. A., Wall A., McGuire A. T., Veesler D. (2020). Cell.

[cit66] Hoffmann M., Kleine-Weber H., Schroeder S., Kruger N., Herrler T., Erichsen S., Schiergens T. S., Herrler G., Wu N. H., Nitsche A., Muller M. A., Drosten C., Pohlmann S. (2020). Cell.

[cit67] Yan R., Zhang Y., Li Y., Xia L., Guo Y., Zhou Y. (2020). Science.

[cit68] Smith M. C., Gestwicki J. E. (2012). Expert Rev. Mol. Med..

[cit69] Josephson K., Ricardo A., Szostak J. W. (2014). Drug Discovery Today.

[cit70] Zhang G., Pomplun S., Loftis A. R., Loas A., Pentelute B. L. (2020). bioRxiv.

[cit71] Wan Y., Shang J., Graham R., Baric R. S., Li F. (2020). J. Virol..

[cit72] Du Q., Wang S., Wei D., Sirois S., Chou K. C. (2005). Anal. Biochem..

[cit73] Han Y., Král P. (2020). ACS Nano.

[cit74] Leader B., Baca Q., Golan D. (2008). Nat. Rev. Drug Discovery.

[cit75] Xia S., Liu Q., Wang Q., Sun Z., Su S., Du L., Ying T., Lu L., Jiang S. (2014). Virus Res..

[cit76] Zhou P., Yang X. L., Shi Z. L. (2020). Nature.

[cit77] Xia S., Zhu Y., Liu M., Lan Q., Xu W., Wu Y., Ying T., Liu S., Shi Z., Jiang S., Lu L. (2020). Cell. Mol. Immunol..

[cit78] Lu G., Wang Q., Gao G. F. (2015). Trends Microbiol..

[cit79] Xia S., Yan L., Xu W., Agrawal A. S., Algaissi A., Tseng C. T. K., Wang Q., Du L., Tan W., Wilson I. A., Jiang S., Yan B., Lu L. (2019). Sci. Adv..

[cit80] Xia S., Liu M., Wang C., Xu W., Lan Q., Feng S., Qi F., Bao L., Du L., Liu S., Qin C., Sun F., Shi Z., Zhu Y., Jiang S., Lu L. (2020). Cell Res..

[cit81] Li G., Clercq E. D. (2020). Nat. Rev. Drug Discovery.

[cit82] Savarino A., Di Trani L., Donatelli I., Cauda R., Cassone A. (2006). Lancet Infect. Dis..

[cit83] Vincent M. J., Bergeron E., Benjannet S., Erickson B. R., Rollin P. E., Ksiazek T. G., Seidah N. G., Nichol S. T. (2005). Virol. J..

[cit84] Rosa S. G. V., Santos W. C. (2020). Rev. Panam. Salud Publica.

[cit85] Wang M., Cao R., Zhang L., Yang X., Liu J., Xu M., Shi Z., Hu Z., Zhong W., Xiao G. (2020). Cell Res..

[cit86] Gautret P., Lagier J. C., Parola P., Meddeb L., Mailhe M., Doudier B., Courjon J., Giordanengo V., Vieira V. E., Dupont H. T., Honoré S. (2020). Int. J. Antimicrob. Agents.

[cit87] Uyeki T. M. (2018). Clin. Infect. Dis..

[cit88] Huang I. C., Li W., Sui J., Marasco W., Choe H., Farzan M. (2008). J. Virol..

[cit89] Wang D., Hu B., Hu C., Zhu F., Liu X., Zhang J., Wang B., Xiang H., Cheng Z., Xiong Y., Zhao Y. (2020). JAMA.

[cit90] Blaising J., Polyak S. J., Pécheur E. I. (2014). Antiviral Res..

[cit91] Shi L., Xiong H., He J., Deng H., Li Q., Zhong Q., Hou W., Cheng L., Xiao H., Yang Z. (2007). Arch. Virol..

[cit92] Borrell B. (2020). Science.

[cit93] Tanaka T., Hishitani Y., Ogata A. (2014). Biol.: Targets Ther..

[cit94] Scott A. M., Allison J. P., Wolchok J. D. (2012). Cancer Immun..

[cit95] Chen X., Li R., Pan Z., Qian C., Yang Y., You R., Zhao J., Liu P., Gao L., Li Z., Huang Q. (2020). Cell. Mol. Immunol..

[cit96] Researching antibodies to target COVID-19, https://www.astrazeneca.com/media-centre/articles/2020/researching-antibodies-to-target-covid-19.html, accessed on May 16, 2020

[cit97] Celltrion , Amgen join race to develop anti SARS-CoV-2 antibodies, https://www.centerforbiosimilars.com/news/celltrion-amgen-join-race-to-develop-antisarscov2-antibodies, accessed on May 16, 2020

[cit98] Regeneron's COVID-19 response efforts, https://www.regeneron.com/covid19, accessed on May 16, 2020

[cit99] Huang C., Wang Y., Li X., Ren L., Zhao J., Hu Y., Zhang L., Fan G., Xu J., Gu X., Cheng Z. (2020). lancet.

[cit100] Xu X., Han M., Li T., Sun W., Wang D., Fu B., Zhou Y., Zheng X., Yang Y., Li X., Zhang X. (2020). Proc. Natl. Acad. Sci. U. S. A..

[cit101] Wan Y., Shang J., Sun S., Tai W., Chen J., Geng Q., He L., Chen Y., Wu J., Shi Z., Zhou Y. (2020). J. Virol..

[cit102] Convalescent Plasma: NIH clinical center, https://www.cc.nih.gov/blooddonor/donationtypes/convalescent_plasma.html, accessed on May 20,2020

[cit103] Zhang J. S., Chen J. T., Liu Y. X., Zhang Z. S., Gao H., Liu Y., Wang X., Ning Y., Liu Y. F., Gao Q., Xu J. G. (2005). J. Med. Virol..

[cit104] Casadevall A., Pirofski L. A. (2020). J. Clin. Invest..

[cit105] Mair-Jenkins J., Saavedra-Campos M., Baillie J. K., Cleary P., Khaw F. M., Lim W. S., Makki S., Rooney K. D., Convalescent Plasma Study Group, Nguyen-Van-Tam J. S., Beck C. R. (2015). J. Infect. Dis..

[cit106] Menis M., Sridhar G., Selvam N., Ovanesov M. V., Divan H. A., Liang Y., Scott D., Golding B., Forshee R., Ball R., Anderson S. A. (2013). Am. J. Hematol..

[cit107] Soo Y. O. Y., Cheng Y., Wong R., Hui D. S., Lee C. K., Tsang K. K. S., Ng M. H. L., Cheng P., Chan G., Sung J. J. Y. (2004). Clin. Microbiol. Infect..

[cit108] Cheng Y., Xing R., Soo Y. O. Y., Wong W. S., Lee C. K., Ng M. H. L., Chan P., Wong K. C., Leung C. B., Cheng G. (2005). Eur. J. Clin. Microbiol..

[cit109] Shen C., Wang Z., Zhao F., Yang Y., Li J., Yuan J., Wang F., Li D., Yang M., Xing L., Wei J. (2020). JAMA.

[cit110] Duan K., Liu B., Li C., Zhang H., Yu T., Qu J., Zhou M., Chen L., Meng S., Hu Y., Peng C. (2020). Proc. Natl. Acad. Sci. U. S. A..

[cit111] Zhang B., Liu S., Tan T., Huang W., Dong Y., Chen L., Chen Q., Zhang L., Zhong Q., Zhang X., Zou Y. (2020). Chest.

[cit112] Hung I. F., To K. K., Lee C. K., Lee K. L., Chan K., Yan W. W., Liu R., Watt C. L., Chan W. M., Lai K. Y., Koo C. K. (2011). Clin. Infect. Dis..

[cit113] Yeh K. M., Chiueh T. S., Siu L. K., Lin J. C., K Chan P., Peng M. Y., Wan H. L., Chen J. H., Hu B. S., Perng C. L., Lu J. J. (2005). J. Antimicrob. Chemother..

[cit114] Ko J. H., Seok H., Cho S. Y., Ha Y. E., Baek J. Y., Kim S. H., Kim Y. J., Park J. K., Chung C. R., Kang E. S., Cho D. (2018). Antiviral Ther..

[cit115] Robbins J. B., Schneerson R., Szu S. C. (1995). J. Infect. Dis..

[cit116] Samuel C. E. (2001). Clin. Microbiol. Rev..

[cit117] Liu Y. J. (2005). Annu. Rev. Immunol..

[cit118] Sen G. C. (2001). Annu. Rev. Microbiol..

[cit119] Sheahan T. P., Sims A. C., Leist S. R., Schäfer A., Won J., Brown A. J., Montgomery S. A., Hogg A., Babusis D., Clarke M. O., Spahn J. E. (2020). Nat. Commun..

[cit120] Chan J. F. W., Yao Y., Yeung M. L., Deng W., Bao L., Jia L., Li F., Xiao C., Gao H., Yu P., Cai J. P. (2015). J. Infect. Dis..

[cit121] Stockman L. J., Bellamy R., Garner P. (2006). PLoS Med..

[cit122] Al-Tawfiq J. A., Momattin H., Dib J., Memish Z. A. (2014). Int. J. Infect. Dis..

[cit123] Shen K. L., Yang Y. H. (2020). World J. Pediatr..

[cit124] Frieman M., Yount B., Heise M., Kopecky-Bromberg S. A., Palese P., Baric R. S. (2007). J. Virol..

[cit125] Kopecky-Bromberg S. A., Martínez-Sobrido L., Frieman M., Baric R. A., Palese P. (2007). J. Virol..

[cit126] Hensley L. E., Fritz E. A., Jahrling P. B., Karp C., Huggins J. W., Geisbert T. W. (2004). Emerging Infect. Dis..

[cit127] Bellingan G., Maksimow M., Howell D. C., Stotz M., Beale R., Beatty M., Walsh T., Binning A., Davidson A., Kuper M., Shah S. (2014). Lancet Respir. Med..

[cit128] Ranieri V. M., Pettilä V., Karvonen M. K., Jalkanen J., Nightingale P., Brealey D., Mancebo J., Ferrer R., Mercat A., Patroniti N., Quintel M. (2020). JAMA.

[cit129] Meng Z., Wang T., Li C., Chen X., Li L., Qin X., Li H., Luo J. (2020). medRxiv.

[cit130] Menachery V. D., Yount B. L., Josset L., Gralinski L. E., Scobey T., Agnihothram S., Katze M. G., Baric R. S. (2014). J. Virol..

[cit131] Siddiqi H. K., Mehra M. R. (2020). J. Heart Lung Transplant..

[cit132] Zhang W., Zhao Y., Zhang F., Wang Q., Li T., Liu Z., Wang J., Qin Y., Zhang X., Yan X., Zeng X. (2020). J. Clin. Immunol..

[cit133] Mantlo E., Bukreyeva N., Maruyama J., Paessler S., Huang C. (2020). Antiviral Res..

[cit134] Zhou Q., Wei X. S., Xiang X., Wang X., Wang Z. H., Chen V., Shannon C. P., Tebbutt S. J., Kollmann T. R., Fish E. N. (2020). medRxiv.

[cit135] Andreakos E., Tsiodras S. (2020). EMBO Mol. Med..

[cit136] Koltsida O., Hausding M., Stavropoulos A., Koch S., Tzelepis G., Übel C., Kotenko S. V., Sideras P., Lehr H. A., Tepe M., Klucher K. M. (2011). EMBO Mol. Med..

[cit137] Yang Z. Y., Kong W. P., Huang Y., Roberts A., Murphy B. R., Subbarao K., Nabel G. J. (2004). Nature.

[cit138] Wu A., Peng Y., Huang B., Ding X., Wang X., Niu P., Meng J., Zhu Z., Zhang Z., Wang J., Sheng J. (2020). Cell Host Microbe.

[cit139] Liu C., Zhou Q., Li Y., Garner L. V., Watkins S. P., Carter L. J., Smoot J., Gregg A. C., Daniels A. D., Jervey S., Albaiu D. (2020). ACS Cent. Sci..

[cit140] Kranz L. M., Diken M., Haas H., Kreiter S., Loquai C., Reuter K. C., Meng M., Fritz D., Vascotto F., Hefesha H., Grunwitz C. (2016). Nature.

[cit141] Islam M. A., Reesor E. K., Xu Y., Zope H. R., Zetter B. R., Shi J. (2015). Biomater. Sci..

[cit142] Schnee M., Vogel A. B., Voss D., Petsch B., Baumhof P., Kramps T., Stitz L. (2016). PLoS Neglected Trop. Dis..

[cit143] Gandhi R. T., Kwon D. S., Macklin E. A., Shopis J. R., McLean A. P., McBrine N., Peter T., Flynn L., Sbrolla A., Kaufmann D. E., Porichis F. (2016). J. Acquired Immune Defic. Syndr..

[cit144] Richner J. M., Himansu S., Dowd K. A., Butler S. L., Salazar V., Fox J. M., Julander J. G., Tang W. W., Shresta S., Pierson T. C., Ciaramella G. (2017). Cell.

[cit145] Cohen J. (2020). Science.

[cit146] National Institutes of Health , NIH clinical trial of investigational vaccine for COVID-19 begins, https://www.nih.gov/news-events/news-releases/nih-clinical-trial-investigational-vaccine-covid-19-begins, accessed on May 16, 2020

[cit147] Chroboczek J., Szurgot I., Szolajska E. (2014). Acta Biochim. Pol..

[cit148] Kushnir N., Streatfield S. J., Yusibov V. (2012). Vaccine.

[cit149] Lan J., Deng Y., Song J., Huang B., Wang W., Tan W. (2018). Virol. Sin..

[cit150] Wang C., Zheng X., Gai W., Zhao Y., Wang H., Wang H., Feng N., Chi H., Qiu B., Li N., Wang T. (2017). Oncotarget.

[cit151] Novavax advances development of novel COID-19 vaccine, http://ir.novavax.com/news-releases/news-release-details/novavax-advances-development-novel-covid-19-vaccine, accessed on May 16, 2020

[cit152] Wang N., Shang J., Jiang S., Du L. (2020). Virol. Sin..

[cit153] Prompetchara E., Ketloy C., Palaga T. (2020). Asian Pac. J. Allergy Immunol..

[cit154] Kam Y. W., Kien F., Roberts A., Cheung Y. C., Lamirande E. W., Vogel L., Chu S. L., Tse J., Guarner J., Zaki S. R., Subbarao K. (2007). Vaccine.

[cit155] GSK and Sanofi join forces to fight COVID-19, https://www.gsk.com/en-gb/media/resource-centre/our-contribution-to-the-fight-against-2019-ncov/sanofi-and-gsk-join-forces-to-fight-covid-19/, accessed on May 16, 2020

[cit156] Bhamarapravati N., Sutee Y. (2000). Vaccine.

[cit157] Sharma A., Krause A., Worgall S. (2011). Hum. Vaccines.

[cit158] Menachery V. D., Gralinski L. E., Mitchell H. D., Dinnon K. H., Leist S. R., Yount B. L., McAnarney E. T., Graham R. L., Waters K. M., Baric R. S. (2018). J. Virol..

[cit159] Codagenix and serum institute of India initiate co-development of a scalable live attenuated vaccine against the COVID-19, https://codagenix.com/news/codagenix-and-serum-institute-of-india-initiate-co-development-of-a-scalable-live-attenuated-vaccine-against-the-2019-novel-coronavirus-covid-19/, accessed on May 16, 2020

[cit160] Jiang S., He Y., Liu S. (2005). Emerging Infect. Dis..

[cit161] Taylor D. (1999). J. Hosp. Infect..

[cit162] He Y., Zhou Y., Siddiqui P., Jiang S. (2004). Biochem. Biophys. Res. Commun..

[cit163] Agrawal S. A., Tao X., Algaissi A., Garron T., Narayanan K., Peng B., Couch R., Tseng K. C. (2016). Hum. Vaccines Immunother..

[cit164] Gao Q., Bao L., Mao H., Wang L., Xu K., Li Y., Zhu L., Wang N., Lv Z., Gao H., Ge X. (2020). bioRxiv.

[cit165] Wang Q., Zhang L., Kuwahara K., Li L., Liu Z., Li T., Zhu H., Liu J., Xu Y., Xie J., Morioka H. (2016). ACS Infect. Dis..

[cit166] Bahl K., Senn J. J., Yuzhakov O., Bulychev A., Brito L. A., Hassett K. J., Laska M. E., Smith M., Almarsson Ö., Thompson J., Ribeiro A. M. (2017). Mol. Ther..

[cit167] Liu M. A. (2019). Vaccines.

[cit168] Prompetchara E., Ketloy C., Palaga T. (2020). Asian Pac. J. Allergy Immunol..

[cit169] Shang W., Yang Y., Rao Y., Rao X. (2020). npj Vaccines.

[cit170] Prompetchara E., Ketloy C., Palaga T. (2020). Asian Pac. J. Allergy Immunol..

[cit171] Amanat F., Krammer F. (2020). Immunity.

[cit172] Agrawal S. A., Tao X., Algaissi A., Garron T., Narayanan K., Peng B., Couch R., Tseng K. C. (2016). Hum. Vaccines Immunother..

[cit173] Xiong S., Wang Y. F., Zhang M. Y., Liu X. J., Zhang C. H., Liu S. S., Qian C. W., Li J. X., Lu J. H., Wan Z. Y., Zheng H. Y. (2004). Immunol. Lett..

[cit174] Prompetchara E., Ketloy C., Palaga T. (2020). Asian Pac. J. Allergy Immunol..

[cit175] Yong C. Y., Ong H. K., Yeap S. K., Ho K. L., Tan W. S. (2019). Virol. Sin..

[cit176] Zhao P., Ke J. S., Qin Z. L., Ren H., Zhao L. J., Yu J. G., Gao J., Zhu S. Y., Qi Z. T. (2004). Acta Biochim. Biophys. Sin..

[cit177] Hasson S. S. A. A., Al-Busaidi J. K. Z., Sallam T. A. (2015). Asian Pac. J. Trop. Biomed..

[cit178] Sampangi-Ramaiah M. H., Vishwakarma R., Shaanker R. U. (2020). Curr. Sci..

[cit179] Ul Qamar M. T., Alqahtani S. M., Alamri M. A., Chen L. L. (2020). J. Pharm. Anal..

[cit180] Redeploying plant defences, Nat. Plants, 2020, 177(6), 10.1038/s41477-020-0628-0 PMC7091691 32170291

[cit181] Balkrishna A., Pokhrel S., Singh J., Varshney A. (2020). Research Square.

[cit182] Ashwagandha takes the lead in recent study to be COVID 19 warrior (May 19, 2020), https://www.thehindubusinessline.com/news/science/ashwagandha-takes-lead-in-iit-delhi-study-to-be-covid-19-warrior/article31621996.ece, accessed on June 1, 2020

[cit183] WHO has recently lifted the ban on the solidarity trial of HCQ, https://www.who.int/dg/speeches/detail/who-director-general-s-opening-remarks-at-the-media-briefing-on-covid-19---03-june-2020, accessed on June 5, 2020

[cit184] WHO welcomes preliminary results about dexamethasone use in treating critically ill covid-19 patient's, https://www.who.int/news-room/detail/16-06-2020-who-welcomes-preliminary-results-about-dexamethasone-use-in-treating-critically-ill-covid-19-patients, accessed on June 17, 2020

